# Effects of immunomodulatory drugs on depressive symptoms: A mega-analysis of randomized, placebo-controlled clinical trials in inflammatory disorders

**DOI:** 10.1038/s41380-019-0471-8

**Published:** 2019-08-19

**Authors:** Gayle M. Wittenberg, Annie Stylianou, Yun Zhang, Yu Sun, Ashutosh Gupta, P. S. Jagannatha, Dai Wang, Benjamin Hsu, Mark E. Curran, Shahid Khan, MRC ImmunoPsychiatry Consortium, Petra E. Vértes, Rudolf Cardinal, Sylvia Richardson, Gwenael Leday, Tom Freeman, David Hume, Tim Regan, Zhaozong Wu, Carmine Pariante, Annamaria Cattaneo, Patricia Zunszain, Alessandra Borsini, Robert Stewart, David Chandran, Livia Carvalho, Joshua Bell, Luis Henrique Souza-Teodoro, Hugh Perry, Neil Harrison, Declan Jones, Robert B Henderson, Guang Chen, Edward T. Bullmore, Wayne C. Drevets

**Affiliations:** 1 Neuroscience, Janssen Research & Development, LLC, Titusville, NJ USA; 2 Clinical Statistics, GlaxoSmithKline R&D, Stevenage, UK; 3 Clinical Statistics, GlaxoSmithKline R&D, Bangalore, India; 4 Immunology, Janssen Research & Development, LLC, Spring House, PA USA; 5 ImmunoPsychiatry, Immuno-Inflammation Therapeutic Area Unit, GlaxoSmithKline R&D, Stevenage, UK; 6 Neuroscience, Janssen Research & Development, LLC, La Jolla, CA USA; 7 University of Cambridge, Department of Psychiatry, Cambridge Biomedical Campus, Cambridge, UK; 8 Cambridgeshire & Peterborough NHS Foundation Trust, Cambridge, UK; 9 University of Cambridge, Cambridge, UK; 10 MRC Biostatistics Unit, Cambridge, UK; 11 University of Edinburgh, Edinburgh, UK; 12 King’s College London, London, UK; 13 University College London, London, UK; 14 University of Southampton, Southampton, UK; 15 University of Sussex, Brighton, UK; 16 Janssen R&D, London, UK; 17 GlaxoSmithKline, Stevenage, UK

**Keywords:** Depression, Psychology

## Abstract

Activation of the innate immune system is commonly associated with depression. Immunomodulatory drugs may have efficacy for depressive symptoms that are co-morbidly associated with inflammatory disorders. We report a large-scale re-analysis by standardized procedures (mega-analysis) of patient-level data combined from 18 randomized clinical trials conducted by Janssen or GlaxoSmithKline for one of nine disorders (*N* = 10,743 participants). Core depressive symptoms (low mood, anhedonia) were measured by the Short Form Survey (SF-36) or the Hospital Anxiety and Depression Scale (HADS), and participants were stratified into high (*N* = 1921) versus low-depressive strata based on baseline ratings. Placebo-controlled change from baseline after 4–16 weeks of treatment was estimated by the standardized mean difference (SMD) over all trials and for each subgroup of trials targeting one of 7 mechanisms (IL-6, TNF-α, IL-12/23, CD20, COX2, BLγS, p38/MAPK14). Patients in the high depressive stratum showed modest but significant effects on core depressive symptoms (SMD = 0.29, 95% CI [0.12–0.45]) and related SF-36 measures of mental health and vitality. Anti-IL-6 antibodies (SMD = 0.8, 95% CI [0.20–1.41]) and an anti-IL-12/23 antibody (SMD = 0.48, 95% CI [0.26–0.70]) had larger effects on depressive symptoms than other drug classes. Adjustments for physical health outcome marginally attenuated the average treatment effect on depressive symptoms (SMD = 0.20, 95% CI: 0.06–0.35), but more strongly attenuated effects on mental health and vitality. Effects of anti-IL-12/23 remained significant and anti-IL-6 antibodies became a trend after controlling for physical response to treatment. Novel immune-therapeutics can produce antidepressant effects in depressed patients with primary inflammatory disorders that are not entirely explained by treatment-related changes in physical health.

## Introduction

Activation of the innate immune system is associated with major depressive disorder (MDD). Case-control studies and meta-analyses have reported that patients have modestly elevated peripheral blood levels of pro-inflammatory cytokines and acute phase proteins, including C-reactive protein (CRP) [1, 2], tumor necrosis factor-alpha (TNF-α) [3–5], and interleukin-6 (IL-6) [4, 6]. Increased cerebrospinal fluid levels of pro-inflammatory cytokines have been reported [7, 8] and correlated with reduced hippocampal volume in depressed patients [9]. A central pro-inflammatory process has also been indicated by post mortem studies of microglial activation and PET studies of TSPO ligand binding in MDD [10, 11].

Clinical evidence for a causal effect of inflammatory challenge on the pathogenesis of depressive symptoms includes data from interferon alpha (IFN-α) treatment trials for hepatitis C, which frequently induces symptoms of depression and fatigue, with a concomitant increase in inflammatory markers in peripheral blood [12] and CSF [13]. In the chronic social defeat stress model in rodents, animals susceptible to developing persistent depression-like behaviors manifest higher peripheral blood levels of IL-6 both before and after stress exposure [14]. Susceptibility to developing depressive phenotypes was reduced in IL-6^−/−^ animals; but increased in wild type animals by transplanting the immune cells of donor animals that had previously expressed a depressive response to social stress [14].

To date, only a few studies have addressed the therapeutic hypothesis that anti-inflammatory drugs may have antidepressant efficacy. Infliximab, an anti-TNF-α antibody, was not effective for depressive symptoms in subjects with treatment-resistant MDD; but *post hoc* analysis indicated that the subgroup of patients with high CRP was more responsive [15]. A small molecule inhibitor of P38 MAP kinase was not consistently effective in two studies of MDD [16]. However, there was evidence of a moderate-sized anti-inflammatory drug effect (SMD = 0.34; 95% CI [0.11–0.57]) on depressive symptoms in a meta-analysis of clinical trial data on NSAIDs and anti-cytokine antibodies in patients with a primary diagnosis of depression or inflammatory disorder [17], and in a meta-analysis of anti-cytokine antibody effects on depressive symptoms in inflammatory disorders (SMD = 0.44; 95% CI [0.22–0.59]) [4, 18–21].

In any analysis of depressive symptom changes during anti-inflammatory drug treatment of an inflammatory disorder it is important to control for treatment effects on physical symptoms (e.g., swollen and painful joints in rheumatoid arthritis). Anti-inflammatory drug effects on psychological symptoms may arise secondarily to treatment effects on the physical signs and symptoms of inflammatory disorders. Alternatively, the antidepressant efficacy of anti-inflammatory drugs may reflect a direct, mechanistically related effect of treatment. This hypothetical dilemma remains unresolved [17, 18].

Here we report a large, integrated analysis of existing clinical trial datasets to further investigate anti-inflammatory drug effects on depressive symptoms. Access to patient-level data (*N* = 10,743) enabled us to identify the cohort of trial participants with high depressive symptoms at baseline, to focus on improvement in the DSM 5 cardinal depressive symptoms of depressed mood and anhedonia, and to control for physical health outcomes (although high depressive patients were not randomly allocated to treatment groups in the primary studies). We analyzed 18 double-blind, placebo-controlled, randomized clinical trials, sponsored by Janssen or GlaxoSmithKline, of 9 compounds targeting 7 mechanisms of action (TNF-α, IL-12/23, IL-6, CD20, COX2, BLγS, and P38/MAPK14) in patients with a primary diagnosis of one of 9 inflammatory or oncological disorders (see Table 1).

**Table 1:** Characteristics of clinical trials included in the mega-analysis. Placebo-controlled, randomized clinical trials of immunomodulatory drugs for treatment of inflammatory or oncological disorders were included if SF-36 or HADS data on depressive symptoms were available at baseline and a follow-up visit 4–16 weeks after randomization

	Clinical Trial ID	Study Drug	Number of Subjects (% high depressive)	Depressive Symptom Scale	Primary Disease	Primary Disease Symptom Scale	Treatment Arms	Follow-up Visit^‡^
	Janssen Trials							
TNF-α	C0168T37*^#^	Infliximab	358 (20%)	SF-36 v1.0	Ulcerative Colitis	MAYO	Placebo (119:90) 5 mg (119:111), 10 mg (120:108)	8 wk
	C0168T41*^#^	Infliximab	1025 (23%)	SF-36 v1.0	Rheumatoid Arthritis	DAS28-CRP	Placebo (345:306) 3 mg (341:282), 10 mg (339:274)	6 wk
	C0168T44#	Infliximab	832 (13%)	SF-36 v1.0	Psoriasis	PASI	Placebo (208:188) 3 mg (310:302), 5 mg (314:306)	10 wk
	C0524T03#	Golimumab	303 (10%)	SF-36 v1.0	Asthma	FEV1	Placebo (77:70) 50 mg (72:59), 100 mg (76:68), 200 mg (78:66)	12 wk
	C0524T09*	Golimumab	350 (18%)	SF-36 v1.0	Ankylosing Spondylitis	ASAS20	Placebo (76:76) 50 mg (136:131), 100 mg (138:135)	14 wk
IL-12/23	C0743T08*^#^	Ustekinumab	763 (8%)	SF-36 v2.0	Psoriasis	PASI	Placebo (254:252) 45 mg (255:255), 90 mg (254:248)	12 wk
	C0743T09#	Ustekinumab	1219 (27%)	HADS	Psoriasis	PASI	Placebo (405:396) 45 mg (405:401), 90 mg (409:404)	12 wk
IL-6	C1377T04*^#^	Sirukumab	176 (26%)	SF-36 v2.0	Rheumatoid Arthritis	DAS28-CRP	Placebo (45:40) 100 mg/2wk (45:44), 25 mg/4wk (27:27) 50 mg/4wk (29:27), 100 mg/4wk (30:28)	12 wk
	MCD2001*^#^	Siltuximab	77 (20%)	SF-36 v2.0	Multicentric Castleman’s Disease	MCDOS	Placebo (26:25) 11 mg/kg /3wk (51:49)	6 wk
	GlaxoSmithKline Trials							
CD20	OFA110634*	Ofatumumab	161 (34%)	SF-36 v2.0	Rheumatoid Arthritis	DAS28-CRP	Placebo (79:65) 700 mg (82:57)	16 wk
	OFA110635*	Ofatumumab	244 (29%)	SF-36 v2.0	Rheumatoid Arthritis	DAS28-CRP	Placebo (122:112) 700 mg (122:105)	16 wk
Cox2	CXA30007	GW406381	1101 (10%)	SF-36 v2.0	Osteoarthritis-Knee	WOMAC	Placebo (184:133) 1 mg (186:133), 5 mg (186:130), 10 mg (184:131), 25 mg (179:133), 50 mg (181:137)	12 wk
	CXA30009	GW406381	1711 (20%)	SF-36 v2.0	Rheumatoid Arthritis	DAS28CRP	Placebo (341:245) 5 mg (348:266), 10 mg (348:273), 25 mg (344:250), 50 mg (330:244)	12 wk
BlγS	BEL110751*^#^	Belimumab	812 (16%)	SF-36 v2.0	Lupus (SLE)	SELENA SLEDAI	Placebo (273:246), 1 mg (269:248), 10 mg (270:252)	12 wk
	BEL110752*	Belimumab	860 (17%)	SF-36 v2.0	Lupus (SLE)	SELENA SLEDAI	Placebo (288:277) 1 mg (285:274), 10 mg (287:276)	12 wk
	LBS02*^#^	Belimumab	445 (12%)	SF-36 v2.0	Lupus (SLE)	SELENA SLEDAI	Placebo (113:103), 1 mg (114:104), 4 mg (111:104), 10 mg (107:100)	12 wk
P38	KIP112967	Losmapimod	167 (17%)	SF-36 v2.0	Neuropathic Pain	PI-NRS	Placebo (80:67), 7.5 mg (87:72)	4 wk
	KIP113049	Losmapimod	139 (12%)	SF-36 v2.0	Neuropathic Pain	PI-NRS	Placebo (71:68), 7.5 mg (68:65)	4 wk

Annotations: *indicates inclusion in non-responder analysis, ^#^indicates studies with significant treatment effect on primary endpoint (physical disease symptom severity scale), ^‡^follow-up visit indicates the week at which depression improvement was assessed in this study, and not the final endpoint for the study. *MAYO* Mayo Score for Ulcerative Colitis, *DAS28-CRP* Disease Activity Score using C-Reactive Protein, *PASI* Psoriasis Area Severity Index, *FEV1* Forced Expiratory Volume 1, *ASAS20* Assessment In Ankylosing Spondylitis Response Criteria, *MCDOS* Multicentric Castleman’s Disease Overall Score, *WOMAC* Western Ontario and McMaster Universities Arthritis Index, *SELENA SLEDAI* Safety of Estrogens in Lupus Erythematosus National Assessment (SELENA) modification of the SLE (Systemic Lupus Erythematosus) Disease Activity Index (SLEDAI) Score, *PI-NRS* Pain Intensity Numeric Rating Scale. Within the treatment arms column, the number of patients at the baseline line and follow-up visits are indicated in parenthesis as (N_baseline_:N_followup_)

## Methods

### Study inclusion criteria

Placebo-controlled, double-blind, randomized, parallel group studies, with publically pre-registered designs, were included if (1) the drug primarily targeted an immune mechanism of action and (2) depressive symptom severity was assessed at baseline and follow-up visits scheduled 4–16 weeks post-randomization; see Table 1 and Supplementary Table 1 for details.

### Outcome measures

For all but one trial, the SF-36 Health Survey (version 1.0 or 2.0) [22] was used as a patient reported outcome (PRO) measure. The SF-36 comprises 36 self-report measures of physical and mental health that can be summarized by 8 domain scores and two component scores (physical and mental health). We used the mental health component score and the vitality domain score as standard SF-36 outcomes. Additionally, to focus on depressive symptoms, we constructed a depressive symptom summary score (range, 0–100). This was based on the two SF-36 questions (“Have you felt downhearted and depressed?” and “Have you felt so down in the dumps that nothing could cheer you up?”) that most closely corresponded to core DSM-5 symptoms of depressed mood and anhedonia; see Supplementary Information. In one study (C0743T09), the Hospital Anxiety and Depression Scale (HADS [23]) was used instead of the SF-36. In an independent study where both scales were measured, the HADS-D was significantly correlated with the defined SF-36 depressive symptom score (Spearman *r* = 0.63, *p* < 0.0001).

### Depressive symptom stratification

Patients were stratified as belonging to high depressive or low depressive subgroups based on their scores on the two SF-36 questions related to depressed mood and anhedonia. A patient was assigned to the high depressive stratum if they rated at least one of these two key symptoms as present at least “most of the time” in the previous 4 weeks and rated the other symptom as present at least “some of the time”; Supplementary Fig. 1. In C0743T09, patients were classified as high depressive if baseline total HADS score was ≥8 [23]. Notably patients with high depressive symptoms were not randomly allocated to treatment in any of the studies (Supplementary Table 2), which fundamentally constrains causal interpretation of treatment effects on this subgroup of patients.

### Analysis of baseline data and treatment effects

Treatment effects were estimated using mixed-effect linear models with repeated measures (MMRM). MMRMs were chosen for their ability to leverage all available data and to minimize the introduction of biases in the context of missing data under the assumption of missing at random [24]. The extent of missing data for each study, due to participant withdrawal, is indicated in Table 1 by the difference between N at baseline and follow-up assessments. Separate models were fit for patients in high and low depressive symptom strata. Depressive symptom score was the primary dependent variable. Treatment, time, and treatment-by-time interaction were fixed effects with time modeled as a repeated measure. Participants were treated as random effects in the model. For multi-country studies with >35 patients per treatment arm (Table 1), country was included as a covariate. The association of baseline biomarkers with treatment response is shown in Supplementary Table 3. The effects on antidepressant treatment outcomes of age, body mass index (BMI), sex and corticosteroid use were not consistently significant across studies; see Supplementary Table 2 and Supplementary Fig. 2.

The within-treatment change was estimated by contrasting the least square means of depressive symptom score at baseline and first follow-up visits. The drug treatment effect was estimated by contrasting the symptom change in the drug treated arm versus the placebo arm. Analyses were performed using SAS 9.2 and 9.4 (www.sas.com), and R 3.3.0. The statistical framework is described in more detail for the illustrative example of a phase 2 trial of sirukumab for RA, C1377T04 (Supplementary Fig. 3).

### Adjustment for treatment effects on physical health

We controlled the estimation of treatment effects on mental health for the effects of treatment on physical health in two ways: (i) for each study, the severity scale used to measure clinical efficacy for primary disease signs and symptoms (e.g., DAS28-CRP in a rheumatoid arthritis trial C1377T04) was added to the mixed model as a time-dependent fixed effect; (ii) for a subset of 12 studies (delineated in Table 1) that had specified a responder/non-responder criterion *a priori*, we estimated the treatment effect on depressive symptoms only in those high depressive patients who were defined as non-responders on the primary (physical health) endpoint of the trial.

### Mega-analysis

For each study, the standardized mean difference (SMD) was estimated by Cohen’s d: the difference in least square means between the treatment and placebo arms divided by the pooled standard deviation. This unit-less measure can be compared and combined across studies [17]. The R package *metafor* was used for analysis and visualization of forest plots. Treatment effects are reported primarily in terms of 95% confidence intervals on the mean SMD; if the 95% CI does not include zero, the treatment effect is statistically significant with two-tailed *P* < 0.05. Heterogeneity across studies was estimated by tau^2^, I^2^ and Cochran’s Q statistic (see Supplementary Information).

## Results

### Study characteristics

The clinical trials included are listed in Table 1. Active treatment groups were defined as patients receiving the new immunomodulatory drug at any dose. Treatment and placebo groups may have received concomitant medication as detailed in Supplementary Table 1.

**Fig. 1:**
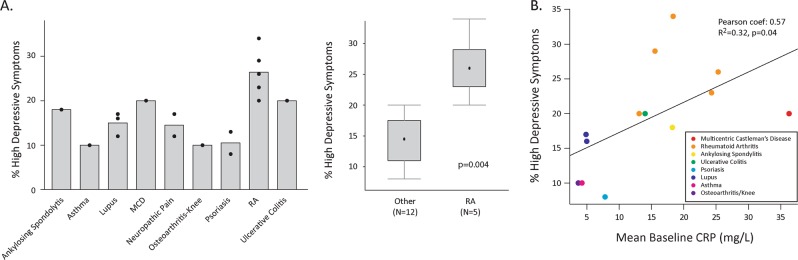
High depressive symptoms in clinical trial participants at baseline. **a** Left panel, percentage of patients meeting criteria for high depressive symptoms at baseline for each trial, grouped by the primary disease treated in the study. Abbreviations: rheumatoid arthritis (RA), multicentric Castleman’s disease (MCD). Right panel, boxplot indicating significantly higher percentage of patients with high depressive symptoms in RA studies compared with other studies combined. The box and whiskers plot indicates median value, interquartile range and extreme values. **b** Scatterplot of percentage of patients with high depressive symptom scores at baseline vs. mean baseline C-reactive protein (CRP). Each point corresponds to a study

For each study we used self-reported measures of mood and anhedonia at baseline to stratify patients into two subgroups, designated as high or low depressive. The proportion of patients belonging to the high depressive stratum varied between studies categorized by primary disorder (Table 1; Fig. 1a), with the greatest proportion of high depressive patients in studies of rheumatoid arthritis (*P* = 0.004, 2-tailed *t*-test, rheumatoid arthritis vs. all other disorders). Baseline CRP was measured in most studies and the mean baseline CRP (averaged across all patients in each study) correlated positively with the proportion of high depressive patients (Pearson *R*^2^ = 0.32, *P* = 0.04, Fig. 1b).

### Treatment and placebo effects on core depressive symptoms

In the high depressive stratum of patients (*N* = 1921 over all 18 studies), we estimated the change from baseline in depressive symptom severity in each treatment arm (active drug or placebo) in each study. Active drug treatment was always associated with significant improvement in depressive symptoms. However, in most (17) studies there was also significant improvement in depressive symptoms after treatment with placebo plus allowed concomitant medication. The placebo effect size varied widely between studies (Supplementary Fig. 4), possibly reflecting the heterogeneity of trial designs with respect to the control of concomitant drugs, like methotrexate or corticosteroids, that are known to affect mood states, or differences in disease states and study populations.

**Fig. 2:**
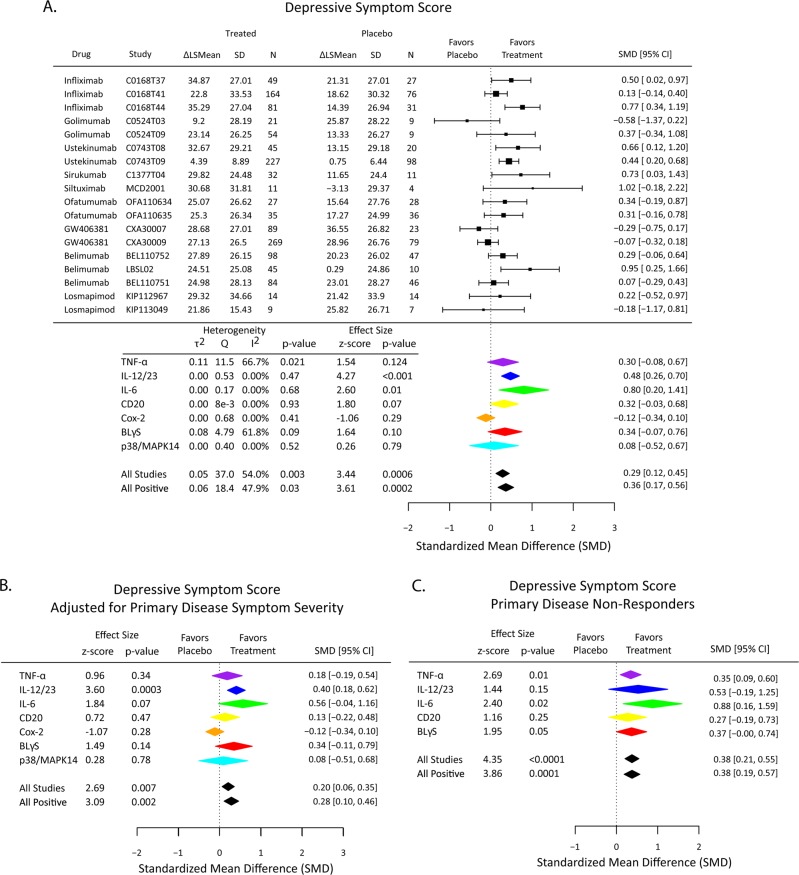
Effects of immunomodulatory drugs (overall and classified by mechanism of action) on depressive symptoms in high depressive stratum of patients. **a** Change in depressive symptom scores from baseline to follow-up visit was compared between active treatment and placebo arms. The standardized mean difference (SMD) is a measure of placebo-controlled antidepressant effect size that can be compared and combined across studies. **b** Immunomodulatory drug effects on depressive symptoms were estimated by a linear model including the primary disease symptom scale appropriate for each study (Table 1) as a covariate to control for drug effects on physical health outcome. **c** Immunomodulatory drug effects on depressive symptoms were estimated only in the subgroup of high depressive patients who did not respond physically to drug treatment (non-responders)

Over all 18 trials, there was a modest but significant antidepressant effect of immunological treatments compared to placebo (SMD = 0.29; 95% CI [0.12,0.45]) (Fig. 2a).

To explore the significant heterogeneity of effect sizes related to mechanistic differences between drugs, we computed placebo-controlled treatment effects on change from baseline depressive symptom scores for each of 7 clusters or classes of studies targeting the same mechanism of action: 5 studies targeted TNFα (3, infliximab; 2, golimumab); 3 targeted BLγS (belimumab); and 2 targeted IL-6 (1, sirukumab; 1, siltuximab) IL-12/23 (ustekinumab), CD20 (ofatumumab), P38/MAPK (losmapimod), or COX-2 (GW406381). The anti-IL-12/23 antibody demonstrated significant improvement in depressive symptoms compared to placebo (SMD = 0.48; 95% CI [0.26, 0.70]). Studies of the two anti-IL-6 antibodies also demonstrated significant antidepressant efficacy vs. placebo (SMD = 0.80; 95% CI [0.20, 1.41]). There were non-significant trends in favor of improved depressive symptoms in patients treated with the anti-BLγS antibody (SMD = 0.34; 95%CI [−0.07, 0.76]), and the two anti-TNFα antibodies (SMD = 0.30; 95% CI [−0.08, 0.67]). Studies of the small molecule COX-2 inhibitor (GW406381) demonstrated a non-significant trend in favor of improved depressive symptoms in patients treated with placebo (SMD = −0.12, 95% CI [−0.34, 0.10]), but it was notable that the change in the placebo arm of this study appeared unusually large (Supplementary Fig. 4).

### Controlling for treatment effects on physical health outcomes

First, we included the continuous measure of physical sign and symptom severity measured for each study as a covariate in the model used to estimate treatment effects on depressive symptoms (Table 1). After this statistical adjustment, antidepressant effects were somewhat attenuated, but the primary mega-analytic estimate of effect size over all studies remained significantly different from zero (SMD = 0.20; 95% CI [0.06, 0.35]). The antidepressant effect of ustekinumab (targeting IL-12/23) remained significant after correction for physical sign and symptom changes (SMD = 0.40; 95% CI [0.18, 0.62]); whereas the antidepressant effect of drugs targeting IL-6 was attenuated to a non-significant trend (Fig. 2b).

Second, using data from 7 Janssen and 5 GSK studies for which a prior decision rule could be used to dichotomize patients as “responders” or “non-responders” with respect to the primary (physical health) endpoint of each trial, we estimated treatment effects on depressive symptoms in the non-responder subgroups alone. The overall antidepressant effect remained significant (SMD = 0.38 95% CI [0.21, 0.55]), with significant effects on depressive symptom severity found in non-responders to treatment with anti-TNF-α (SMD = 0.35; 95% CI [0.09–0.60]) and anti-IL-6 (SMD = 0.88; 95% CI [0.16–1.59]) antibodies (Fig. 2c).

### Effects of treatment on the SF-36 Mental health component score and the vitality domain score

**Fig. 3:**
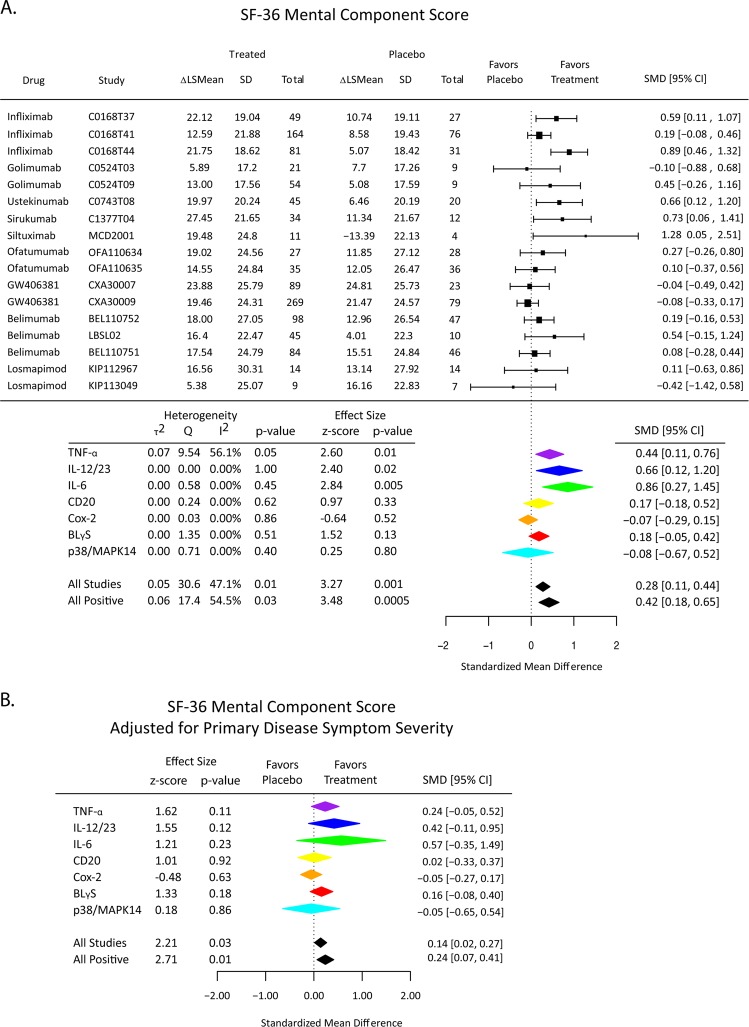
Effects of immunomodulatory drugs (overall and classified by mechanism of action) on SF-36 Mental Health Component (MC) scores in the high depressive stratum of patients. **a** Change in SF-36 MC scores from baseline to follow-up visit was compared between active treatment and placebo arms. The standardized mean difference (SMD) is a measure of placebo-controlled antidepressant effect size that can be compared and combined across studies. **b** Immunomodulatory drug effects on SF-36 MC scores were estimated by a linear model including the primary disease symptom scale appropriate for each study (Table 1) as a covariate to control for drug effects on physical health outcome

**Fig. 4:**
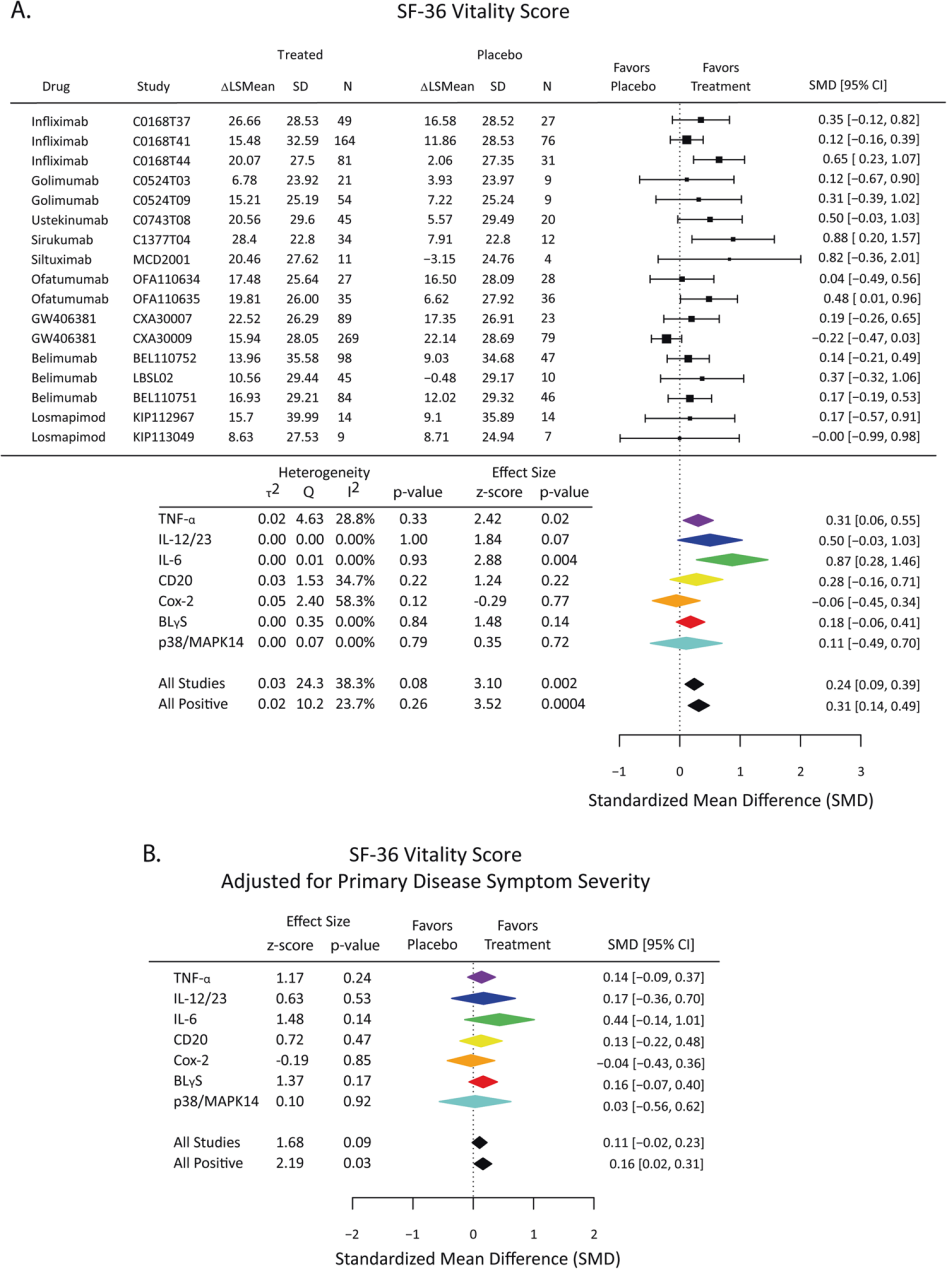
Effects of immunomodulatory drugs (overall and classified by mechanism of action) on SF-36 Vitality Domain scores in the high depressive stratum of patients. **a** Change in SF-36 vitality domain scores from baseline to follow-up visit was compared between active treatment and placebo arms. The standardized mean difference (SMD) is a measure of placebo-controlled antidepressant effect size that can be compared and combined across studies. **b** Immunomodulatory drug effects on SF-36 vitality domain scores were estimated by a linear model including the primary disease symptom scale appropriate for each study (Table 1) as a covariate to control for drug effects on physical health outcome

There was a significant effect of anti-inflammatory drug treatment over the 17 studies reporting the SF-36 for both the mental health component score (SMD = 0.28; 95% CI [0.11, 0.44]) and the vitality domain score (SMD = 0.24; 95% CI [0.09, 0.39]). However, treatment effects on the mental health component score were reduced (SMD = 0.14; 95% CI [0.02, 0.27]), and the vitality domain score was attenuated to a non-significant trend, by statistical control for treatment effects on physical health (Figs. 3 and 4).

### Sensitivity Analyses

There was no significant treatment effect of anti-inflammatory drugs on the depressive symptom severity score, the mental health component score, or the vitality domain score, in parallel analyses of SF-36 data including all subjects, rather than just those with high depressive symptoms (In 18 trials; depressive symptom score: SMD = 0.00; 95% CI [−0.05, 0.06], see Supplementary Figs. 5–7). Additional sensitivity analyses were included to assess the effects of the additional covariates of age, gender, and corticosteroid use (Supplementary Fig. 8 and Supplementary Table 2), and the stringency of the definition for the high depressive symptom cohort (Supplementary Fig. 2). The effects on the SF-36 anhedonia and depression items were evaluated separately, and the effect of treatment on depressive symptoms among primary disease responders is shown (Supplementary Fig. 2).

## Discussion

Our principal findings are that depressive symptoms are frequent among patients recruited to clinical trials for non-psychiatric inflammatory disorders and that immunomodulatory drug treatment generally causes a modest, but significant, improvement in depressive symptoms, specifically in the subgroups of patients with high depressive symptoms at baseline (SMD = 0.29; 95% CI. 0.12–0.45). These results are compatible with prior data implicating inflammation in the pathophysiology and response to treatment of depression [1, 2, 25, 26]. This modest effect size is comparable to meta-analytic estimates of antidepressant efficacy of selective serotonin reuptake inhibitors in patients with major depressive disorder [27], and comparable to the standardized effect sizes seen in meta-analyses of inflammatory cytokines in case-control cohorts [1, 4, 6, 28].

In evaluating these results, it is reasonable to ask how much of the improvement in mental health is attributable to treatment benefits for the primary disease states evaluated in these trials. When we controlled statistically for treatment effects on physical health, the mega-analytic estimate of antidepressant effect size was reduced (SMD = 0.20; 95% CI, 0.06–0.35) but remained significant. Likewise, anti-IL-6 and anti-TNF antibodies had significant beneficial effects on core depressive symptoms, even in patients who had not responded to treatment in terms of improved physical health for the primary disease states studied. In contrast, broader measures of mental health or vitality, which included questions probing somatic symptoms, such as fatigue, were less robust to statistical correction for physical health outcomes. We conclude that the effects of anti-inflammatory drugs on depressive symptoms are not entirely attributable to their effects on physical health. However, it may be that somatic symptoms (e.g., fatigue) are more strongly coupled to peripheral tissue disease activity than psychological symptoms (e.g., anhedonia). Nevertheless, it is noteworthy that conventional assessments of primary disease severity sometimes included a biomarker index of inflammation (e.g., the DAS28-CRP index used to assess rheumatoid arthritis severity includes CRP). Adjusting antidepressant effects of treatment by DAS28-CRP scores may correct for not only physical health effects of treatment but may also attenuate the effect size of any treatment on depressive symptoms or fatigue that are mediated by inflammatory mechanisms.

The antidepressant effect size varied between different classes of drug target. Antibodies targeting IL-6 or IL-12 and IL-23 (IL-12/23) had large and statistically significant effect sizes on core depressive symptoms before correction for physical health outcomes. Moreover, the antidepressant effect of ustekinumab (anti-IL-12/23 antibody) remained significant after correction for physical health outcome, and the effect of sirukumab and siltuximab (anti-IL-6 antibodies) remained significant in non-responders for the primary disease states evaluated. A variety of evidence implicates IL-6 in the pathogenesis of depression [9, 29, 30], and a phase 2 trial of sirukumab for patients with MDD and CRP >3 mg/L has recently completed (clinicaltrials.gov ID: NCT02473289). Increased levels of IL-12 in depressed patients were reduced by monoaminergic antidepressant treatment [31, 32]. Antibodies targeting BLγS and TNF-α also demonstrated trend-level efficacy for depressive symptoms. Small molecules targeting P38MAPK or COX2 demonstrated the least antidepressant effect which is compatible with the lack of compelling evidence for antidepressant efficacy of these mechanisms in previously published MDD trials [16].

The main strength of this study is that we have reported depressive symptom outcomes in 1921 patients treated with one of a range of mechanistically selective and innovative drugs in randomized clinical trials. Access to patient-level data enabled *post hoc* patient stratification and statistical controls for physical health outcomes, which is not possible in literature-based meta-analyses. The main limitations are related to the fact that the primary studies were not prospectively designed to test drug effects on depressive or other psychological states. For example, depressive symptoms were usually assessed by the SF-36 questionnaire. This PRO measure has the merit of being widely used, allowing consistent evaluation of treatment effects across a large number of studies and participants; however, it was not intended to serve as an endpoint for antidepressant efficacy. It is noteworthy, nonetheless, that the depressive symptom score derived from the SF-36 is significantly correlated with HADS-D scores. Similarly, the vitality domain score of the SF-36 includes questions related to fatigue but it is not designed to test efficacy of anti-inflammatory drugs in treating symptoms of fatigue.

A related issue is that the studies were focused on a diverse range of primary disorders. The comparisons between different anti-inflammatory drug effects on depression were not controlled by design for type or severity of physical comorbidity, although we endeavored to mitigate this issue by including physical health measures as covariates in the analysis of depressive symptom scores. The primary studies also varied in terms of the “standard of care” provided to patients in both placebo and active treatment groups. In particular, studies differed in terms of allowed concomitant medications and the percentage of patients using corticosteroids. In each study, however, patients in both the placebo and active treatment groups were subject to the same standard of care, so this potential between-study difference appeared unlikely to bias within-study estimation of treatment effects; furthermore, a *post hoc* analysis found no significant effect of corticosteroid use on between-study variation in treatment effect size (Supplementary Table 2). Likewise, in each study, patients were well-matched for age, sex, and BMI between treatment groups, suggesting that these factors are unlikely to bias estimation of within-study antidepressant effects. We further evaluated the effect of between-study variability in age, sex, and BMI and found only a small age effect indicating that older subjects are less responsive (Supplementary Fig. 2). We were unable to rigorously assess dose-response relationships, because most studies used more than one dose of active treatment, but not always the same dose range in different studies of the same drug, and data on dose/occupancy relationships were not available for all drugs. Finally, patients with high depressive symptoms were not randomly allocated to treatment in any of the studies, which fundamentally constrains causal interpretation of treatment effects in this subgroup of treated patients.

Collectively the limitations of our study highlight the need for future studies designed primarily to evaluate the effects of anti-inflammatory drugs on validated efficacy endpoints for depression and fatigue. Future studies also are needed to explore whether inflammatory biomarkers at baseline can identify subgroups of MDD patients likely to benefit from anti-inflammatory drug treatment. Greater use of predictive biomarkers may also be important in managing safety risks by precluding treatment of patients unlikely to respond, We note that antidepressants include a black box warning indicating they may increase the risk of suicidal thinking in children and adolescents, and that recently the IL-17 inhibitor brodalumab was approved as a treatment for psoriasis with a warning that it has been linked to suicidal ideation [32]. In future trials of immunomodulatory drugs for inflammatory disorders associated with high levels of mental health comorbidity, such as rheumatoid arthritis, it would be useful to measure outcomes early and frequently to test whether direct effects of treatment on mental health can be demonstrated before treatment effects on physical health are evident.

We conclude that anti-inflammatory drugs can have therapeutic effects on psychological symptoms of depression associated with inflammatory disease that are not entirely attributable to treatment effects on physical health. Further studies are required to confirm these findings directly.

## Supplementary information

Supplementary Information

## References

[1] Howren MB, Lamkin DM, Suls J (2009). Associations of depression with C-reactive protein, IL-1, and IL-6: a meta-analysis. Psychosom Med.

[2] Vogelzangs N, Duivis HE, Beekman AT, Kluft C, Neuteboom J, Hoogendijk W (2012). Association of depressive disorders, depression characteristics and antidepressant medication with inflammation. Transl Psychiatry.

[3] Liu Y, Ho RC-M, Mak A (2012). Interleukin (IL)-6, tumour necrosis factor alpha (TNF-α) and soluble interleukin-2 receptors (sIL-2R) are elevated in patients with major depressive disorder: a meta-analysis and meta-regression. J Affect Disord.

[4] Dowlati Y, Herrmann N, Swardfager W, Liu H, Sham L, Reim EK (2010). A meta-analysis of cytokines in major depression. Biol Psychiatry.

[5] Mikova O, Yakimova R, Bosmans E, Kenis G, Maes M (2001). Increased serum tumor necrosis factor alpha concentrations in major depression and multiple sclerosis. Eur Neuropsychopharmacol.

[6] Valkanova V, Ebmeier KP, Allan CL (2013). CRP, IL-6 and depression: a systematic review and meta-analysis of longitudinal studies. J Affect Disord.

[7] Lindqvist D, Janelidze S, Erhardt S, Träskman‐Bendz L, Engström G, Brundin L (2011). CSF biomarkers in suicide attempters–a principal component analysis. Acta Psychiatr Scand.

[8] Lindqvist D, Janelidze S, Hagell P, Erhardt S, Samuelsson M, Minthon L (2009). Interleukin-6 is elevated in the cerebrospinal fluid of suicide attempters and related to symptom severity. Biol Psychiatry.

[9] Frodl T, Carballedo A, Hughes M, Saleh K, Fagan A, Skokauskas N (2012). Reduced expression of glucocorticoid-inducible genes GILZ and SGK-1: high IL-6 levels are associated with reduced hippocampal volumes in major depressive disorder. Transl Psychiatry.

[10] Yirmiya R, Rimmerman N, Reshef R (2015). Depression as a microglial disease. Trends Neurosci.

[11] Setiawan E, Wilson AA, Mizrahi R, Rusjan PM, Miler L, Rajkowska G (2015). Role of translocator protein density, a marker of neuroinflammation, in the brain during major depressive episodes. JAMA Psychiatry.

[12] Bonaccorso S, Puzella A, Marino V, Pasquini M, Biondi M, Artini M (2001). Immunotherapy with interferon-alpha in patients affected by chronic hepatitis C induces an intercorrelated stimulation of the cytokine network and an increase in depressive and anxiety symptoms. Psychiatry Res.

[13] Raison CL, Borisov AS, Majer M, Drake DF, Pagnoni G, Woolwine BJ (2009). Activation of central nervous system inflammatory pathways by interferon-alpha: relationship to monoamines and depression. Biol Psychiatry.

[14] Hodes GE, Pfau ML, Leboeuf M, Golden SA, Christoffel DJ, Bregman D (2014). Individual differences in the peripheral immune system promote resilience versus susceptibility to social stress. Proc Natl Acad Sci USA.

[15] Raison CL, Rutherford RE, Woolwine BJ, Shuo C, Schettler P, Drake DF (2013). A randomized controlled trial of the tumor necrosis factor antagonist infliximab for treatment-resistant depression: the role of baseline inflammatory biomarkers. JAMA Psychiatry.

[16] Inamdar A, Merlo-Pich E, Gee M, Makumi C, Mistry P, Robertson J (2014). Evaluation of antidepressant properties of the p38 MAP kinase inhibitor losmapimod (GW856553) in Major Depressive Disorder: Results from two randomised, placebo-controlled, double-blind, multicentre studies using a Bayesian approach. J Psychopharmacol.

[17] Köhler O, Benros ME, Nordentoft M, Farkouh ME, Iyengar RL, Mors O (2014). Effect of anti-inflammatory treatment on depression, depressive symptoms, and adverse effects: a systematic review and meta-analysis of randomized clinical trials. JAMA Psychiatry.

[18] Kappelmann N, Lewis G, Dantzer R, Jones P B, Khandaker G M (2016). Antidepressant activity of anti-cytokine treatment: a systematic review and meta-analysis of clinical trials of chronic inflammatory conditions. Molecular Psychiatry.

[19] Tyring S, Gottlieb A, Papp K, Gordon K, Leonardi C, Wang A (2006). Etanercept and clinical outcomes, fatigue, and depression in psoriasis: double-blind placebo-controlled randomised phase III trial. Lancet.

[20] Menter A, Augustin M, Signorovitch J, Andrew PY, Wu EQ, Gupta SR (2010). The effect of adalimumab on reducing depression symptoms in patients with moderate to severe psoriasis: a randomized clinical trial. J Am Acad Dermatol.

[21] Langley RG, Feldman SR, Han C, Schenkel B, Szapary P, Hsu M-C (2010). Ustekinumab significantly improves symptoms of anxiety, depression, and skin-related quality of life in patients with moderate-to-severe psoriasis: results from a randomized, double-blind, placebo-controlled phase III trial. J Am Acad Dermatol.

[22] Ware Jr JE (2000). SF-36 health survey update. Spine.

[23] Zigmond AS, Snaith RP (1983). The hospital anxiety and depression scale. Acta Psychiatr Scand.

[24] Gueorguieva R, Krystal JH (2004). Move over ANOVA: Progress in analyzing repeated measures data and its reflection in papers published in the Archives of General Psychiatry. Arch Gen Psychiatry.

[25] Miller AH, Maletic V, Raison CL (2009). Inflammation and its discontents: the role of cytokines in the pathophysiology of major depression. Biol psychiatry.

[26] Raison CL, Capuron L, Miller AH (2006). Cytokines sing the blues: inflammation and the pathogenesis of depression. Trends Immunol.

[27] Fournier JC, DeRubeis RJ, Hollon SD, Dimidjian S, Amsterdam JD, Shelton RC (2010). Antidepressant drug effects and depression severity: a patient-level meta-analysis. Jama.

[28] Köhler CA, Freitas TH, Maes MD, De Andrade NQ, Liu CS, Fernandes BS (2017). Peripheral cytokine and chemokine alterations in depression: a meta‐analysis of 82 studies. Acta Psychiatr Scand.

[29] Maes M, Bosmans E, De Jongh R, Kenis G, Vandoolaeghe E, Neels H (1997). Increased serum IL-6 and IL-1 receptor antagonist concentrations in major depression and treatment resistant depression. Cytokine.

[30] O’Brien SM, Scully P, Fitzgerald P, Scott LV, Dinan TG (2007). Plasma cytokine profiles in depressed patients who fail to respond to selective serotonin reuptake inhibitor therapy. J Psychiatr Res.

[31] Lee K-M, Kim Y-K (2006). The role of IL-12 and TGF-β1 in the pathophysiology of major depressive disorder. Int Immunopharmacol.

[32] Kim Y, Suh I, Kim H, Han C, Lim C, Choi S (2002). The plasma levels of interleukin-12 in schizophrenia, major depression, and bipolar mania: effects of psychotropic drugs. Mol Psychiatry.

