# Supplementary material for: Effects of immunomodulatory drugs on depressive symptoms: A mega-analysis of randomized, placebo-controlled clinical trials in inflammatory disorders
Source: Mol Psychiatry. 2019 Aug 19;25(6):1275–85. doi: 10.1038/s41380-019-0471-8 (PMC7244402; doi:10.1038/s41380-019-0471-8)
Supplement: Supplementary file 1 — Supplementary Information [file 41380_2019_471_MOESM1_ESM.pdf]

SUPPLEMENTARY INFORMATION

Effects of immunomodulatory drugs on depressive symptoms:

A mega-analysis of randomized, placebo-controlled clinical trials in inflammatory disorders

Gayle M. Wittenberg[1], Annie Stylianou[2], Yun Zhang[1], Yu Sun[1], Ashutosh Gupta[3], P. S. Jagannatha[3], Dai Wang[1], Benjamin Hsu[4], Mark E. Curran[4], Shahid Khan[5], MRC ImmunoPsychiatry Consortium, Guang Chen[6], Edward T. Bullmore[5,7,8]*, Wayne C. Drevets[1]*
[1]Neuroscience, Janssen Research & Development, LLC, Titusville, NJ, USA
[2]Clinical Statistics, GlaxoSmithKline R&D, Stevenage, United Kingdom
[3]Clinical Statistics, GlaxoSmithKline R&D, Bangalore, India
[4]Immunology, Janssen Research & Development, LLC, Spring House, PA, USA
[5]ImmunoPsychiatry, Immuno-Inflammation Therapeutic Area Unit, GlaxoSmithKline R&D, Stevenage, United Kingdom
[6]Neuroscience, Janssen Research & Development, LLC, La Jolla, CA, USA
[7]University of Cambridge, Department of Psychiatry, Cambridge Biomedical Campus, Cambridge, United Kingdom
[8] Cambridgeshire & Peterborough NHS Foundation Trust, Cambridge, United Kingdom

Author contributions:

GW, AS, GC, WD, EB, SK, BH, MC: concept development, scientific input. GW, AS, GC, WD, EB: *post hoc* study design, supervision of data analysis, drafting the manuscript. YS, YZ, JPS, AG, AS, DW and GW: statistical methods development, data analysis, manuscript Figures and Tables.

* These authors contributed equally as co-senior authors.

Supplementary Materials

A phenomenological challenge in evaluating depressive symptoms in the context of patients with a comorbid inflammatory disorder or cancer is that some of the DSM V criteria used to establish the diagnosis of MDD, such as fatigue, may be associated with the patients' primary disease. Here we explore (1) the full SF-36 Mental Health Component Score, which includes multiple items expected to be at least partly driven by the primary disease, (2) a depressive symptom score derived based on selection of the two items that match the DSM V core depressive symptoms of "depressed mood" and "anhedonia", (3) the SF-36 Vitality domain score. These were calculated as follows.

SF-36 Mental Health Component Score: This is comprised of 14 questions clustered in four domains of mental function: vitality, social functioning, role-emotional, and mental health. The component score was calculated by inverting individual item scores as needed so that larger scores consistently indicated an improvement in mental health, summing all 14 item scores and scaling the aggregate score from 0 (worst possible mental health score) to 100 (best possible mental health score).

Depressive Symptom Score: The two cardinal depressive symptoms of the DSM 5 diagnostic criteria for MDD, namely depressed mood and anhedonia, were assessed by the following items of the SF-36 Health Survey [1]: "Have you felt downhearted and depressed?" and "Have you felt so down in the dumps that nothing could cheer you up?". Different versions of the SF-36 have rated the response to these questions on slightly different scales. SF-36 v1 was scored with responses "All of the Time"=0, "Most of the Time"=1, "A Good Bit of the Time"=2, "Some of the Time"=3, "A Little of the Time"=4, or "None of the Time"=5. In SF-36 v2, the response "A Good Bit of the Time" has been removed, and the maximum score per item is 4. To allow aggregation of SF-36 data collected on different versions of the questionnaire, item scores were summed and scaled from 0 to 100 to create the depressive symptom score. For SF-36 v1: depressive symptom score = 100*(MH02+MH04)/10. For SF-36 v2: depressive symptom score = 100*(MH02+MH04)/8. An increase in the score corresponds to an improvement in depressive symptoms. In one study (C0743T09), the Hospital Anxiety and Depression Scale (HADS23) was used instead of the SF-36. We analyzed the Depression Score. To define patients as having high depressive symptoms, a cutoff of >= 8 was applied[2,3].

SF-36 Vitality Domain Score: Fatigue-related symptoms are common in MDD and many non-psychiatric disorders. The SF-36 vitality domain score was used as a self-reported measure of fatigue calculated from 4 questionnaire items asking about subjective experience of energy and tiredness. Item responses were inverted as appropriate to ensure that higher scores always denoted higher energy, summed and scaled from 0-100.

Example Statistical Analysis:

In **Supplementary Figure 3**, we walk through an example of the analysis performed using the C1377T04 sirukumab Trial. First, patients were separated into two groups: those with high depressive symptoms at baseline, and those without. As the majority of patients did not exhibit high depressive symptoms, and those symptoms were measured on a scale that saturates at 100, pooling all patients together would dilute a treatment-related effect on changes in depressive symptoms (**Supplementary Figure 3, left**). Separate mixed models were fit in patient groups with high depressive or low-depressive symptoms at baseline. Dependent variables were the Depressive Symptom score, the SF-36 Mental Health Component or the SF-36 Vitality score. Treatment, visit, and treatment-by-visit interaction were fixed-effects with visit included as a repeated measure. To account for changes in symptoms of the primary disease, the DAS28-CRP was added as a time-dependent fixed-effect (**Supplementary Figure 3, right**). The treatment effect was assessed by contrasting the improvement in the treatment arm and the placebo arm. A p-value threshold of 0.05 was used to declare statistical significance. The significance of the change in depressive symptoms is shown, as is the significance of the treatment vs. placebo comparison as described in the methods; which for this study is significant. The changes in the mean scores for the treated and placebo arms, were designated ΔM_T and ΔM_P, respectively.

Heterogeneity of pooled studies

Tests for heterogeneity examine the null hypothesis that all studies are examining the same effect. The heterogeneity of the studies was estimated by Cochran's Q statistic, I^2 and tau^2 [4].

Cochran's Q is calculated $= \sum_{i=1}^{k} W_i (Y_i - M)^2$, which represents the weighted sum of squared deviations between each study's estimate from the pooled estimate. W_i corresponds to the weight of each study, Y_i to the effect size for each study, and M the pooled estimate of effect size.

$I^2 = 100*(Q-df)/Q$, represents the percentage of total variation across studies due to heterogeneity (range 0-100%, with 0% indicating no observed heterogeneity.

$tau^2 = (Q-df)/C$, where $C = \sum_{i=1}^{k} W_i - \frac{\sum_{i=1}^{k} W_i^2}{\sum_{i=1}^{k} W_i}$ estimates the between study variance in a random effects meta-analysis.

Cytokine Biomarkers

A striking contrast between the studies analyzed here and a typical clinical trial in MDD subjects is the high level of inflammatory biomarkers observed in all subjects (**Figure 1B, Supplementary Table 3**). This is not surprising since most patients are undergoing treatment for diagnosed inflammatory disorders. For example, in MDD studies, CRP thresholds of ≥ 3 or ≥ 5 mg/l have been considered for selecting patients with high levels of inflammation [5]. In epidemiological studies of MDD, median measurements of CRP have been in the range of 1.5-3 mg/l [6,7]. Effectively all subjects analyzed here have high levels of inflammatory proteins. Interestingly, compared across all studies, the mean CRP level among patients entering the study correlated with the percentage of patients within that study who manifested high depressive symptoms (**Figure 1B**).

Comparisons were made between patient groups with and without high depressive symptoms at baseline for baseline levels of serum biomarkers using then Wilcoxon rank sum test. Correlations between baseline serum biomarkers and change in depressive symptom score were assessed using (partial) Spearman correlation coefficients adjusted for baseline disease severity. (**Supplementary Table 3**)

References

1. Ware Jr JE. SF-36 health survey update. Spine 2000;25:3130-9.

2. Wilkinson MJB and Barczac P. Psychiatric screening in general practice: comparison of the general health questionnaire and the hospital anxiety depression scale. J R Coll Gen Pract 1988; 38(312):311-313.
3. Brennan, C., Worrall-Davies, A., McMillan, D., Gilbody, S., & House, A. (2010). The Hospital Anxiety and Depression Scale: a diagnostic meta-analysis of case-finding ability. Journal of psychosomatic research, 69(4), 371-378.
4. Borenstein M, Hedges LV, Higgins JPT, Rothstein HR. Overview. Introduction to Meta-Analysis: John Wiley & Sons, Ltd; 2009:103-6.
5. Raison CL, Rutherford RE, Woolwine BJ, et al. A randomized controlled trial of the tumor necrosis factor antagonist infliximab for treatment-resistant depression: the role of baseline inflammatory biomarkers. JAMA psychiatry 2013;70:31-41.
6. Howren MB, Lamkin DM, Suls J. Associations of depression with C-reactive protein, IL-1, and IL-6: a meta-analysis. Psychosomatic medicine 2009;71:171-86.
7. Vogelzangs N, Duivis HE, Beekman AT, et al. Association of depressive disorders, depression characteristics and antidepressant medication with inflammation. Translational psychiatry 2012;2:e79.

Supplementary Figure 1. Definition and calculation of the depressive symptom score from two items of the SF-36 and high depressive symptom criteria.
Supplementary Figure 2. Sensitivity Analyses.
Supplementary Figure 3. Example of analysis of a single data set, the phase 2 study of sirukumab for rheumatoid arthritis.
Supplementary Figure 4. Effectors of change in depressive symptom score
Supplementary Figure 5. Results of anti-inflammatory intervention on depressive symptoms: overall, and by targeted mechanism in all subjects.
Supplementary Figure 6. Results of anti-inflammatory intervention on mental health component score: overall, and by targeted mechanism in all subjects.
Supplementary Figure 7. Results of anti-inflammatory intervention on SF-36 vitality domain score: overall, and by targeted mechanism in all subjects.
Supplementary Figure 8. Effect of mean study covariate values on overall study effect size on depressive symptoms.
Supplementary Table 1: Additional clinical trial details.
Supplementary Table 2: Patient demographics and concomitant corticosteroid use for each trial.
Supplementary Table 3: Baseline biomarkers**.**

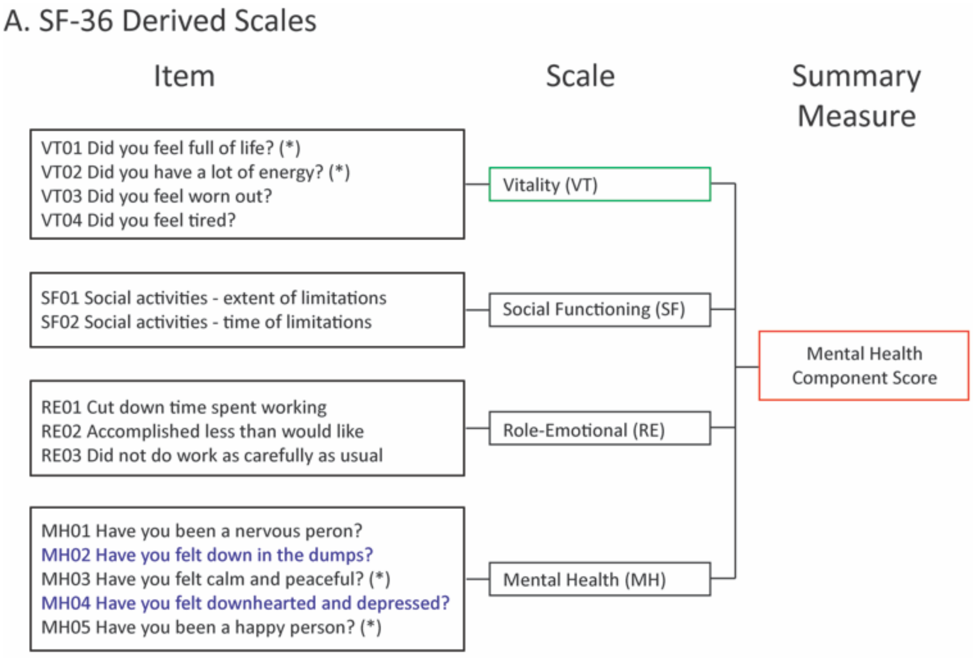

B. Item Ratings

During the past 4 weeks, how often have you experiened symptom:

SF-36 v1.0

5 None of the time
4 A little bit of the time
3 Some of the time
2 A good bit of the time
1 Most of the time
0 All of the time

SF-36 v2.0

4 None of the time
3 A little bit of the time
2 Some of the time
1 Most of the time
0 All of the time

C. High Depressive Symptom Cohort

SF-36 v1.0

MH02: Down in the dumps	MH04: Downhearted and depressed			
	1	2	3	4
1	Yes	Yes	Yes	Yes
2	Yes	Yes	Yes	Yes
3	Yes	Yes	Yes	Yes
4	Yes	Yes	Yes	

SF-36 v2.0

MH02: Down in the dumps	MH04: Downhearted and depressed		
	1	2	3
1	Yes	Yes	Yes
2	Yes	Yes	Yes
3	Yes	Yes	

* For C0743T09, HADS-D >= 8 was used as the cutoff

Supplementary Figure 1. A) Definition and calculation of the depressive symptom score from two items of the SF-36. Depressive symptom score items are indicated in blue (MH02, MH04), the vitality score is shown in green, and the full mental health component score in red. B) Scoring of SF-36 v1.0 and v2.0 C) High depressive symptom criteria by scale.

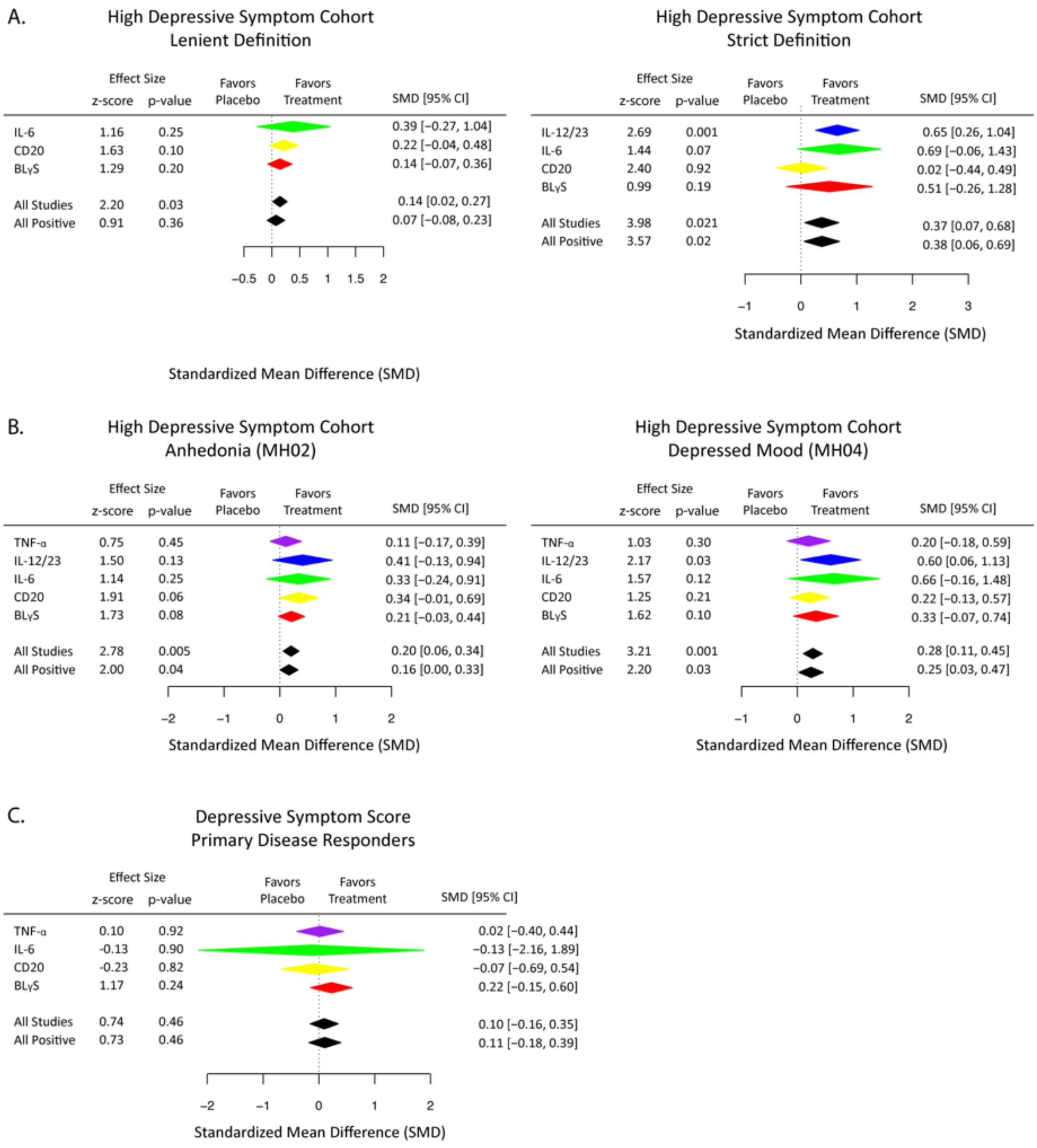

Supplementary Figure 2. Sensitivity Analyses. Positive results from the main analysis in Figure 2 for patients with high depressive symptoms were evaluated for A) The effect of the definition for “High Depressive Symptoms”, evaluating more lenient (left) and stricter (right) definitions where data were available, as described in the Supplementary Methods. B) The individual effects were shown on the two items on the SF-36 composing the depressive symptom score to assess differences in effect on anhedonia (left) and depressed mood (right). C) The effect on depressive symptom score on primary disease responders in the “High Depressive Symptom” cohort is included as a corollary to Figure 2C.

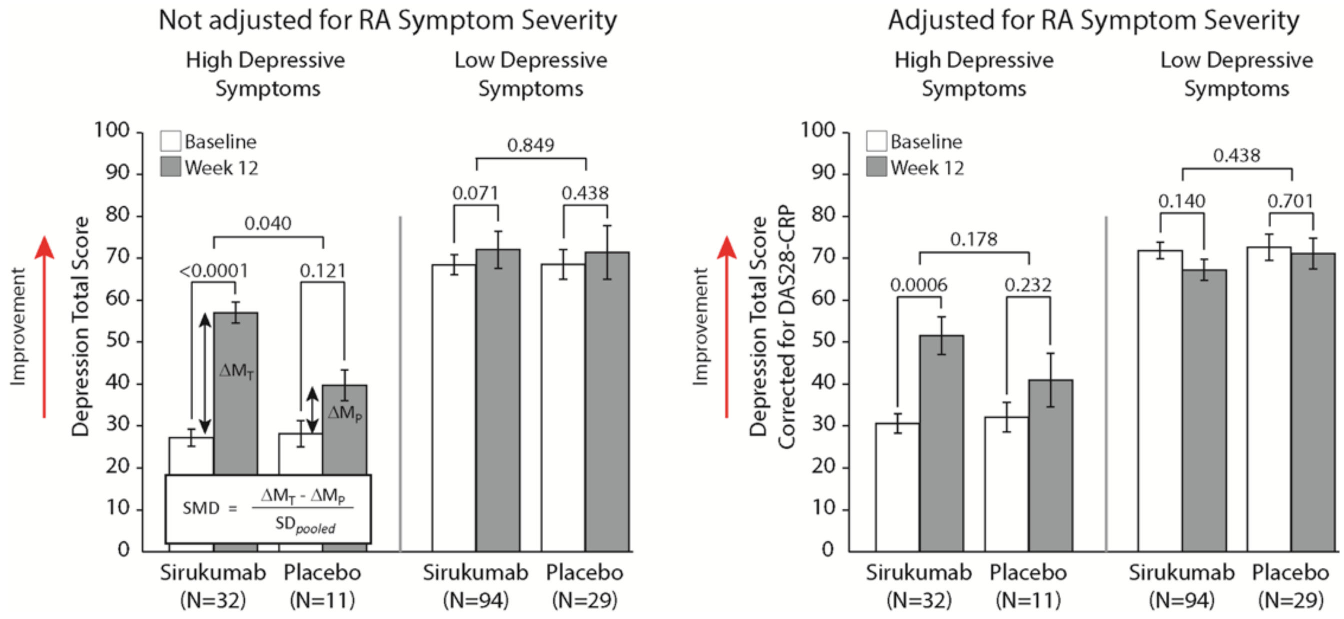

Supplementary Figure 3. Example of analysis of a single data set, the phase 2 study of sirukumab for rheumatoid arthritis. Depression total score at baseline and 12 weeks in the treated and placebo arms for (left) patients without high depressive symptoms at baseline and (right) patients with high depressive symptoms at baseline. The mean difference in the placebo (ΔM_P) and treated (ΔM_T) arms, used in the calculation of the standardized mean difference, is indicated. Error bars are SEM.

A.

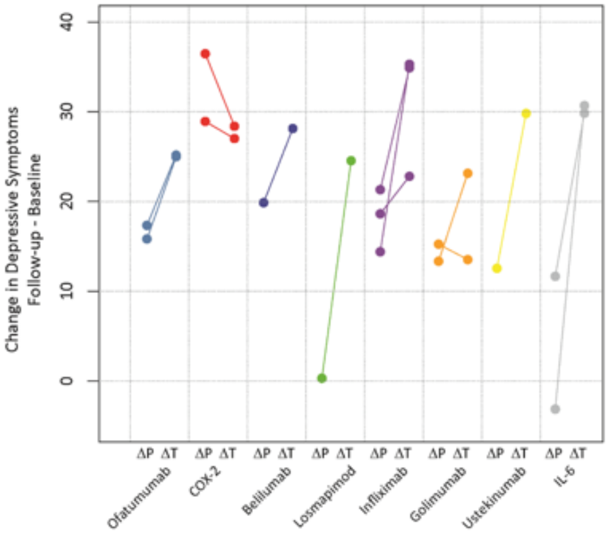

B.

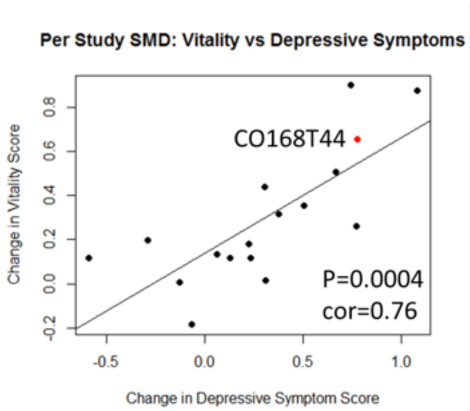

C.

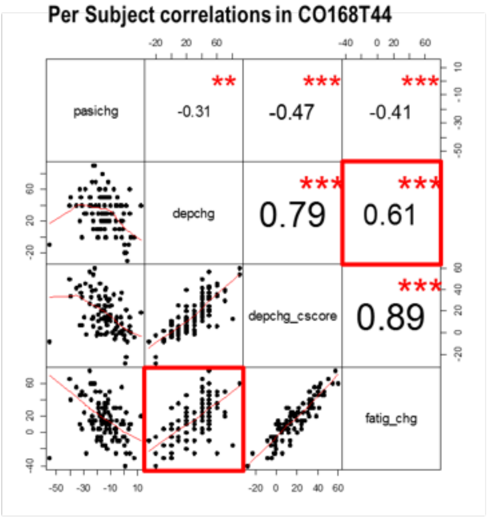

*** p-value <0.001

** p-value <0.01

Supplementary Figure 4. Effectors of change in depressive symptom score. A. The change in depressive symptom score is calculated as the difference between the score at the follow-up timepoint and the baseline score for each study. This is calculated for the placebo arm and the pooled treatment arms for each study. Lines connect changes in placebo and treatment arms from the same study. Studies are grouped by mechanism of action. B. Comparison of placebo ΔM_P and treated ΔM_T is shown for each study, grouped by drug. Connected points correspond to data from the same trial. Data from the ustekinumab trial using the HADS score are not shown due to the different scale. C. Within treated arms, for each study (1 point = 1 study), change in depressive symptom score is plotted versus the change in vitality score. D. Correlations between changes in raw PASI score (pasichg), depressive symptom score (depchg), mental health

component score (depchg_cscore), and vitality score (fatig_chg), for all participants in study CO168T44.

A.

Depressive Symptom Score
All subjects

Drug	Study	Treated ΔLSMean	Treated SD	Treated Total	Placebo ΔLSMean	Placebo SD	Placebo Total	SMD [95% CI]
Infliximab	C0168T37	10.45	44.08	239	8.42	36.19	119	0.05 [−0.17 , 0.27]
Infliximab	C0168T41	7.11	38.19	680	4.07	34.97	345	0.08 [−0.05 , 0.21]
Infliximab	C0168T44	7.89	50.84	624	−0.06	37.3	208	0.17 [0.01 , 0.32]
Golimumab	C0524T03	0.82	41.25	227	0.19	33.52	77	0.02 [−0.24 , 0.27]
Golimumab	C0524T09	7.66	55.56	274	0.39	38.84	76	0.14 [−0.12 , 0.39]
Ustekinumab	C0743T08	4.33	57.52	509	−0.82	45.98	254	0.10 [−0.06 , 0.25]
Ustekinumab	C0743T09	1.87	12.3	814	−0.21	9.43	405	0.18 [0.06 , 0.30]
Sirukumab	C1377T04	10.27	33.05	131	5.32	32.89	45	0.15 [−0.19 , 0.49]
Siltuximab	MCD2001	7.89	39.18	25	−1.58	38.03	26	0.24 [−0.31 , 0.79]
Ofatumumab	OFA110634	11.77	42.63	82	3.5	43.4	79	0.19 [−0.12, 0.50]
Ofatumumab	OFA110635	10.63	44.43	122	4.82	45.21	122	0.13 [−0.12, 0.38]
GW406381	CXA30007	4.9	55.96	917	7.97	55.82	184	−0.05 [−0.21, 0.10]
GW406381	CXA30009	−5.53	53.99	1370	10.01	53.64	341	−0.29 [−0.41, −0.17]
Belimumab	BEL110752	7.42	34.43	572	4.43	34.42	288	0.09 [−0.05, 0.23]
Belimumab	LBSL02	1.54	34.38	332	0.27	34.29	113	0.04 [−0.18, 0.25]
Belimumab	BEL110751	4.44	46.16	539	4.63	45.68	273	−0.00 [−0.15, 0.14]
Losmapimod	KIP112967	5.87	56.8	87	3.47	54.93	80	0.04 [−0.26, 0.35]
Losmapimod	KIP113049	5.41	33.01	68	2.22	33.82	71	0.09 [−0.24, 0.43]

	Heterogeneity τ^2	Q	I^2	p-value	Effect Size z-score	p-value	SMD [95% CI]
TNF-α	0.00	1.46	0.00%	0.83	2.39	0.02	0.10 [0.02, 0.18]
IL-12/23	0.00	0.78	0.00%	0.38	3.11	0.002	0.15 [0.05, 0.24]
IL-6	0.00	0.08	0.00%	0.78	1.18	0.24	0.17 [−0.11, 0.46]
CD20	0.00	0.09	0.00%	0.76	1.55	0.12	0.15 [−0.04, 0.35]
Cox-2	0.02	5.33	81.2%	0.021	-1.52	0.13	−0.18 [−0.41, 0.05]
BLγS	0.00	0.77	0.00%	0.68	0.89	0.37	0.04 [−0.05, 0.13]
p38/MAPK14	0.00	0.05	0.00%	0.82	0.58	0.56	0.07 [−0.16, 0.29]
All Studies	0.01	43.2	56.2%	0.0005	1.56	0.12	0.06 [−0.01, 0.12]
All Positive	0.00	5.50	0.00%	0.79	3.73	0.0002	0.10 [0.05, 0.15]

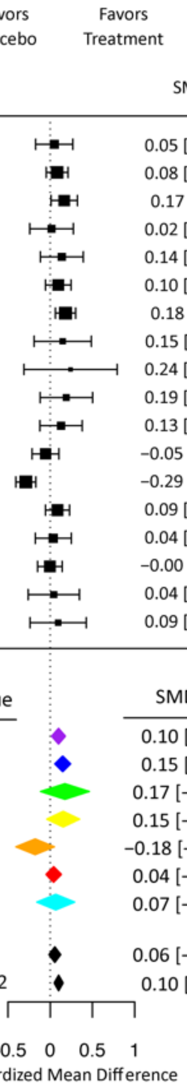

B.

Depressive Symptom Score - All Subjects
Adjusted for Primary Disease Symptom Severity

	Effect Size z-score	p-value	SMD [95% CI]
TNF-α	0.80	0.42	0.03 [−0.05, 0.11]
IL-12/23	0.93	0.35	0.04 [−0.05, 0.14]
IL-6	0.03	0.97	0.01 [−0.28, 0.29]
CD20	0.42	0.68	0.04 [−0.15, 0.24]
Cox-2	-1.27	0.20	−0.15 [−0.39, 0.08]
BLγS	0.77	0.44	0.04 [−0.06, 0.13]
p38/MAPK14	0.45	0.65	0.05 [−0.17, 0.28]
All Studies	0.19	0.85	0.00 [−0.05, 0.06]
All Positive	1.34	0.18	0.04 [−0.02, 0.09]

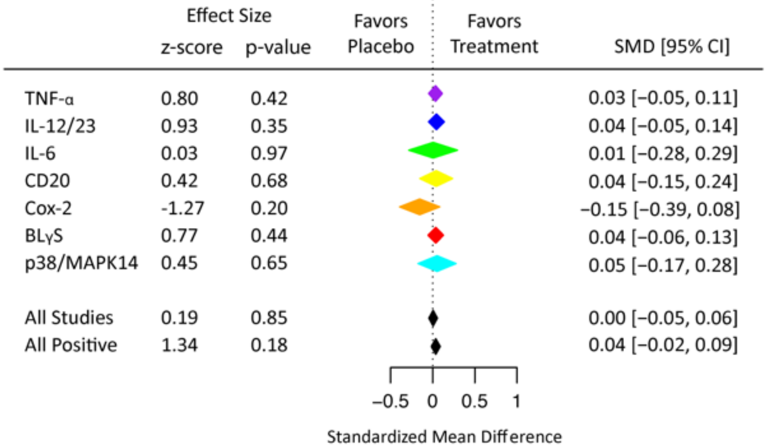

Supplementary Figure 5. Results of anti-inflammatory intervention on depressive symptoms: overall, and by targeted mechanism in all subjects. A. Change in depressive symptoms score from baseline to follow-up visit is compared across treated and placebo arms. The standardized mean difference is used to compare trials on an equivalent metric and compare by mechanism. B. A mixed effects model with repeated measures was fit, as described in the Methods, including the primary disease symptom scale indicated in **Table 1** as an additional repeated measure.

A.

SF-36 Mental Health Component Score
All subjects

Drug	Study	Treated ΔLSMean	Treated SD	Treated Total	Placebo ΔLSMean	Placebo SD	Placebo Total	SMD [95% CI]
Infliximab	C0168T37	12.82	31.82	239	9.18	25.91	119	0.12 [−0.10 , 0.34]
Infliximab	C0168T41	7.8	26.34	680	3.14	24.06	345	0.18 [0.05 , 0.31]
Infliximab	C0168T44	7.83	36.35	624	−0.59	26.44	208	0.25 [0.09 , 0.40]
Golimumab	C0524T03	2.93	31.14	227	0.57	24.18	77	0.08 [−0.18 , 0.34]
Golimumab	C0524T09	8.6	36.59	274	0.39	25.63	76	0.24 [−0.02 , 0.49]
Ustekinumab	C0743T08	3.95	41.78	509	−1.65	33.33	254	0.14 [−0.01 , 0.29]
Sirukumab	C1377T04	13.91	25.39	131	9.43	25.24	45	0.18 [−0.16 , 0.52]
Siltuximab	MCD2001	8.63	29.04	50	−1.35	28.26	26	0.34 [−0.13 , 0.82]
Ofatumumab	OFA110634	11.49	38.86	82	2.52	39.47	79	0.23 [−0.08, 0.54]
Ofatumumab	OFA110635	9.00	33.72	122	3.4	35.19	122	0.16 [−0.09, 0.41]
GW406381	CXA30007	5.16	30.68	917	5.62	30.38	184	−0.02 [−0.17, 0.14]
GW406381	CXA30009	8.04	36.45	1370	8.1	36.35	341	−0.00 [−0.12, 0.12]
Belimumab	BEL110752	6.5	32.66	574	4.34	32.64	288	0.07 [−0.08, 0.21]
Belimumab	LBSL02	1.18	29.04	335	2.02	28.97	113	−0.03 [−0.24, 0.18]
Belimumab	BEL110751	4.2	40.21	542	4.07	39.75	274	0.00 [−0.14, 0.15]
Losmapimod	KIP112967	3.03	36.69	87	3.08	36.10	80	−0.00 [−0.30, 0.30]
Losmapimod	KIP113049	3.17	27.92	68	2.35	28.64	71	0.03 [−0.30, 0.36]

	Heterogeneity τ^2	Q	I^2	p-value	Effect Size z-score	p-value	SMD [95% CI]
TNF-α	0.00	1.70	0.00%	0.79	4.48	<0.0001	0.19 [0.10, 0.27]
IL-12/23	0.00	0.00	0.00%	1.00	1.86	0.06	0.14 [−0.01, 0.29]
IL-6	0.00	0.31	0.00%	0.58	1.65	0.10	0.23 [−0.04, 0.51]
CD20	0.00	0.11	0.00%	0.75	1.89	0.06	0.19 [−0.01, 0.38]
Cox-2	0.00	0.18	0.00%	0.89	-0.13	0.89	−0.01 [−0.10, 0.09]
BLγS	0.00	0.65	0.00%	0.72	0.51	0.61	0.02 [−0.07, 0.12]
p38/MAPK14	0.00	0.02	0.00%	0.90	0.11	0.91	0.01 [−0.21, 0.24]
All Studies	0.002	17.1	20.3%	0.38	3.52	0.0004	0.10 [0.04, 0.15]
All Positive	0.001	7.43	12.7%	0.49	3.93	0.0001	0.13 [0.06, 0.19]

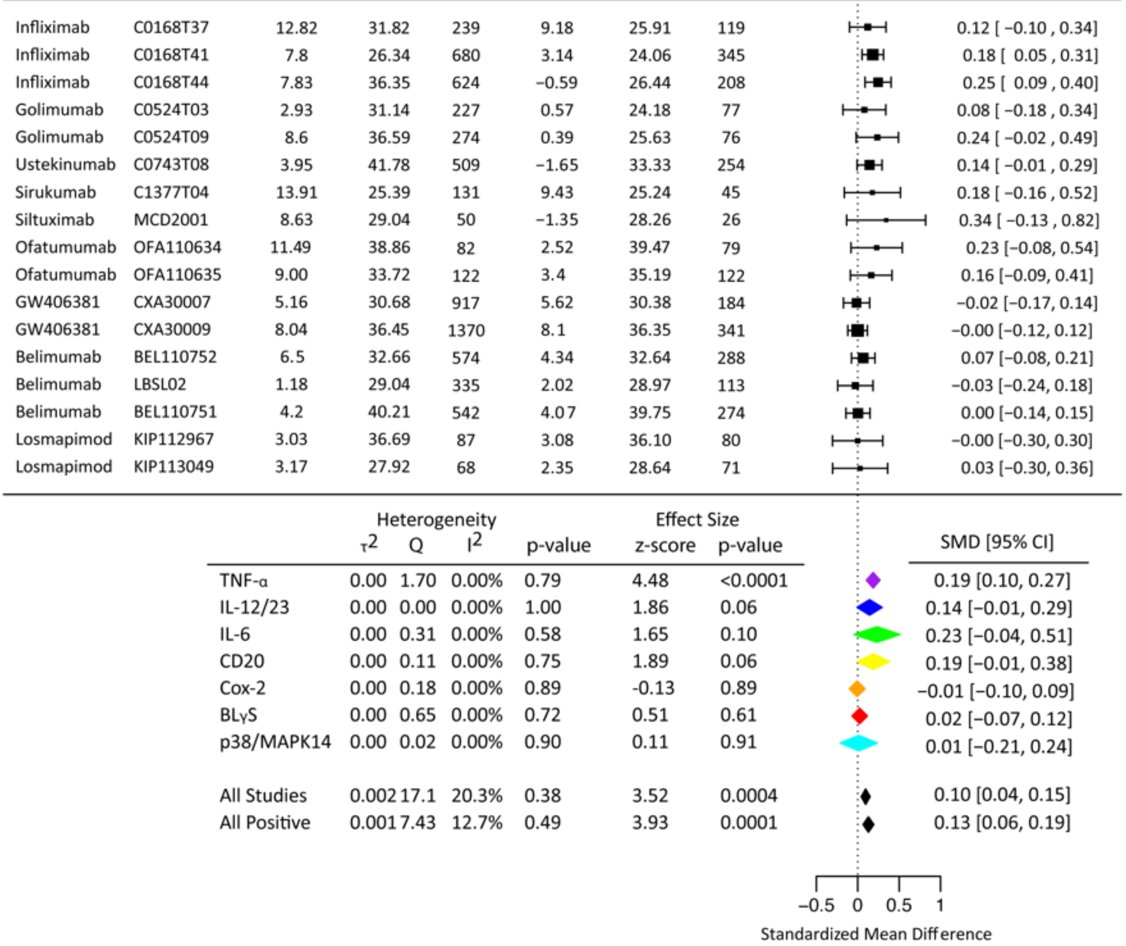

B.

SF-36 Mental Health Component Score - All Subjects
Adjusted for Primary Disease Symptom Severity

	Effect Size z-score	p-value	SMD [95% CI]
TNF-α	1.88	0.06	0.08 [−0.00, 0.16]
IL-12/23	0.05	0.96	0.00 [−0.15, 0.15]
IL-6	0.30	0.76	0.07 [−0.37, 0.51]
CD20	0.73	0.47	0.07 [−0.12, 0.27]
Cox-2	-0.40	0.69	−0.02 [−0.11, 0.08]
BLγS	0.35	0.73	0.02 [−0.08, 0.11]
p38/MAPK14	-0.13	0.90	−0.01 [−0.24, 0.21]
All Studies	1.22	0.22	0.03 [−0.02, 0.07]
All Positive	1.60	0.11	0.05 [−0.01, 0.11]

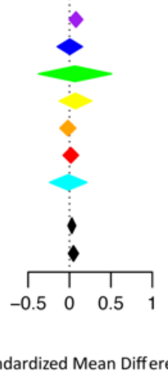

Supplementary Figure 6. Results of anti-inflammatory intervention on mental health component score: **overall, and by targeted mechanism in all subjects.** A. Change in depressive symptoms score from baseline to follow-up visit is compared across treated and placebo arms. The standardized mean difference is used to compare trials on an equivalent metric and compare by mechanism. B. A mixed effects model with repeated measures was fit, as described in the Methods, including the primary disease symptom scale indicated in **Table 1** as an additional repeated measure.

A.

SF-36 Vitality domain score
All subjects

Drug	Study	Treated ΔLSMean	Treated SD	Treated Total	Placebo ΔLSMean	Placebo SD	Placebo Total	SMD [95% CI]
Infliximab	C0168T37	19.45	45.88	239	14.87	37.34	119	0.11 [−0.11 , 0.33]
Infliximab	C0168T41	11.6	35.35	680	6	32.26	345	0.16 [0.03 , 0.29]
Infliximab	C0168T44	8.62	52.15	624	−0.77	37.86	208	0.19 [0.03 , 0.35]
Golimumab	C0524T03	4.43	41.83	227	1.38	33.9	77	0.08 [−0.18 , 0.33]
Golimumab	C0524T09	13.76	51.59	274	1.71	36.61	76	0.25 [−0.01 , 0.50]
Ustekinumab	C0743T08	3.32	60.49	509	−3.25	48.2	254	0.12 [−0.03 , 0.27]
Sirukumab	C1377T04	16.43	26.2	131	11.24	26.13	45	0.20 [−0.14 , 0.54]
Siltuximab	MCD2001	11.55	33.17	51	0.13	32.23	26	0.34 [−0.13 , 0.82]
Ofatumumab	OFA110634	14.47	38.88	82	5.89	39.48	79	0.22 [−0.09, 0.53]
Ofatumumab	OFA110635	15.71	30.22	122	3.29	31.53	122	0.40 [0.15, 0.65]
GW406381	CXA30007	8.39	32.36	917	8.07	32.05	184	0.01 [−0.15, 0.17]
GW406381	CXA30009	10.38	35.27	1370	10.62	35.47	341	−0.01 [−0.13, 0.11]
Belimumab	BEL110752	8.16	52.36	577	5.8	52.18	288	0.05 [−0.10, 0.19]
Belimumab	LBSL02	4.6	29.99	335	3.29	30.01	113	0.04 [−0.17, 0.26]
Belimumab	BEL110751	8.05	42.39	542	6.06	41.91	274	0.05 [−0.10, 0.19]
Losmapimod	KIP112967	1.76	44.42	87	3.34	43.69	80	−0.04 [−0.34, 0.27]
Losmapimod	KIP113049	8.46	31.78	68	1.46	32.58	71	0.22 [−0.12, 0.55]

	Heterogeneity τ^2	Q	I^2	p-value	Effect Size z-score	p-value	SMD [95% CI]
TNF-α	0.00	1.24	0.00%	0.87	3.92	0.0001	0.16 [0.08, 0.24]
IL-12/23	0.00	0.00	0.00%	1.00	1.51	0.13	0.12 [−0.03, 0.27]
IL-6	0.00	0.24	0.00%	0.62	1.75	0.08	0.25 [−0.03, 0.52]
CD20	0.00	0.80	0.00%	0.37	3.27	0.001	0.33 [0.13, 0.52]
Cox-2	0.00	0.03	0.00%	0.87	0.02	0.99	−0.00 [−0.10, 0.09]
BLγS	0.00	0.00	0.00%	1.00	0.98	0.33	0.05 [−0.05, 0.14]
p38/MAPK14	0.01	1.12	16.6%	0.27	0.64	0.52	0.08 [−0.17, 0.33]
All Studies	0.002	17.8	14.4%	0.34	4.03	0.0001	0.10 [0.05, 0.16]
All Positive	0.00	4.42	0.00%	0.82	3.86	0.0001	0.12 [0.06, 0.17]

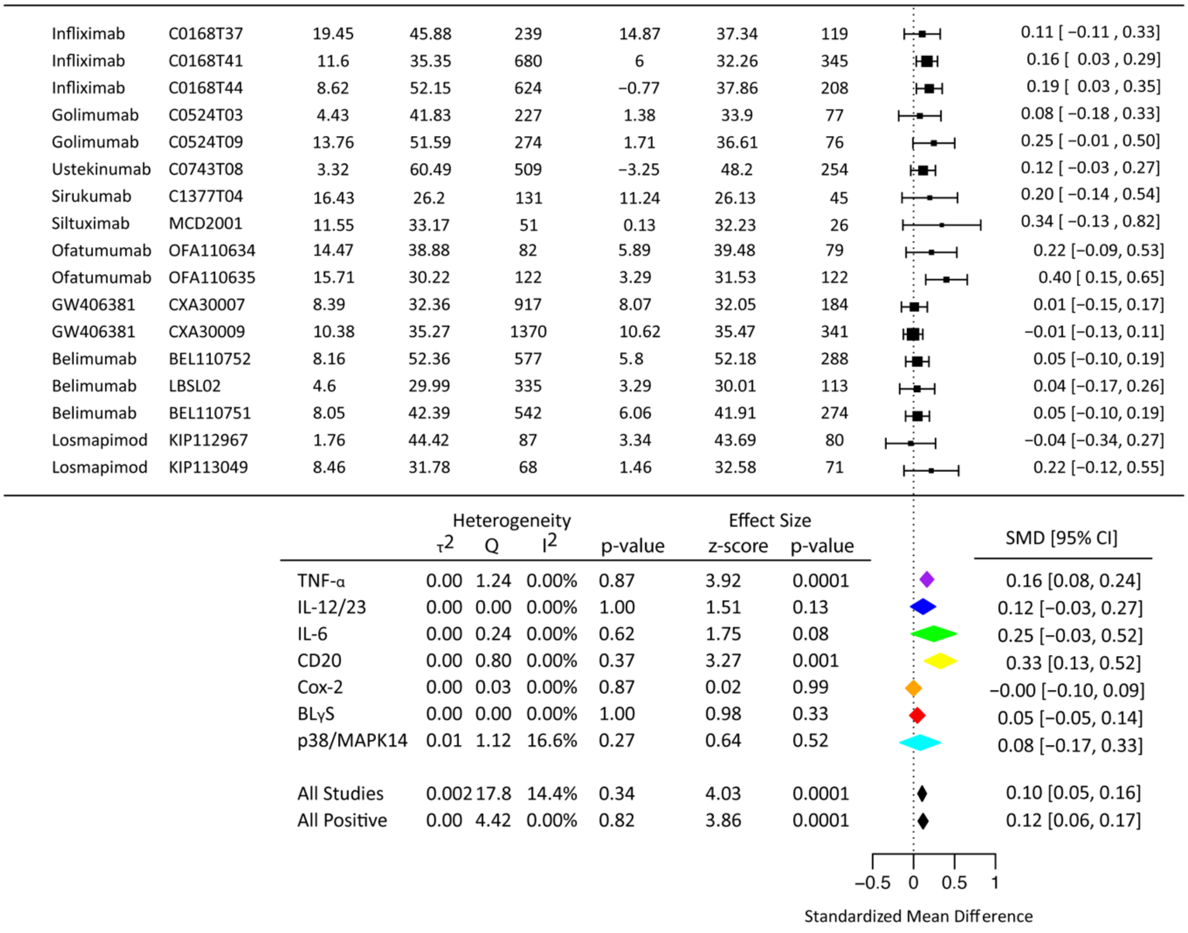

B.

SF-36 Vitality Domain Score - All Subjects
Adjusted for Primary Disease Symptom Severity

	Effect Size z-score	p-value	SMD [95% CI]
TNF-α	1.53	0.13	0.06 [−0.02, 0.14]
IL-12/23	0.02	0.98	−0.00 [−0.15, 0.15]
IL-6	0.32	0.75	0.07 [−0.36, 0.50]
CD20	1.83	0.07	0.18 [−0.01, 0.38]
Cox-2	-0.24	0.81	−0.01 [−0.11, 0.08]
BLγS	0.84	0.40	0.04 [−0.05, 0.13]
p38/MAPK14	0.48	0.63	0.06 [−0.18, 0.30]
All Studies	1.72	0.09	0.04 [−0.01, 0.09]
All Positive	1.45	0.15	0.04 [−0.02, 0.10]

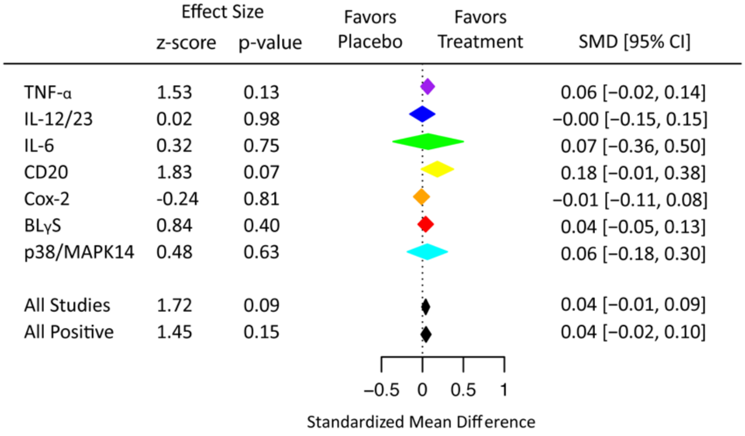

Supplementary Figure 7. Results of anti-inflammatory intervention on SF-36 vitality domain score: **overall, and by targeted mechanism in all subjects.** A. Change in depressive symptoms score from baseline to follow-up visit is compared across treated and placebo arms. The standardized mean difference is used to compare trials on an equivalent metric and compare by mechanism. B. A mixed effects model with repeated measures was fit, as described in the Methods, including the primary disease symptom scale indicated in **Table 1** as an additional repeated measure.

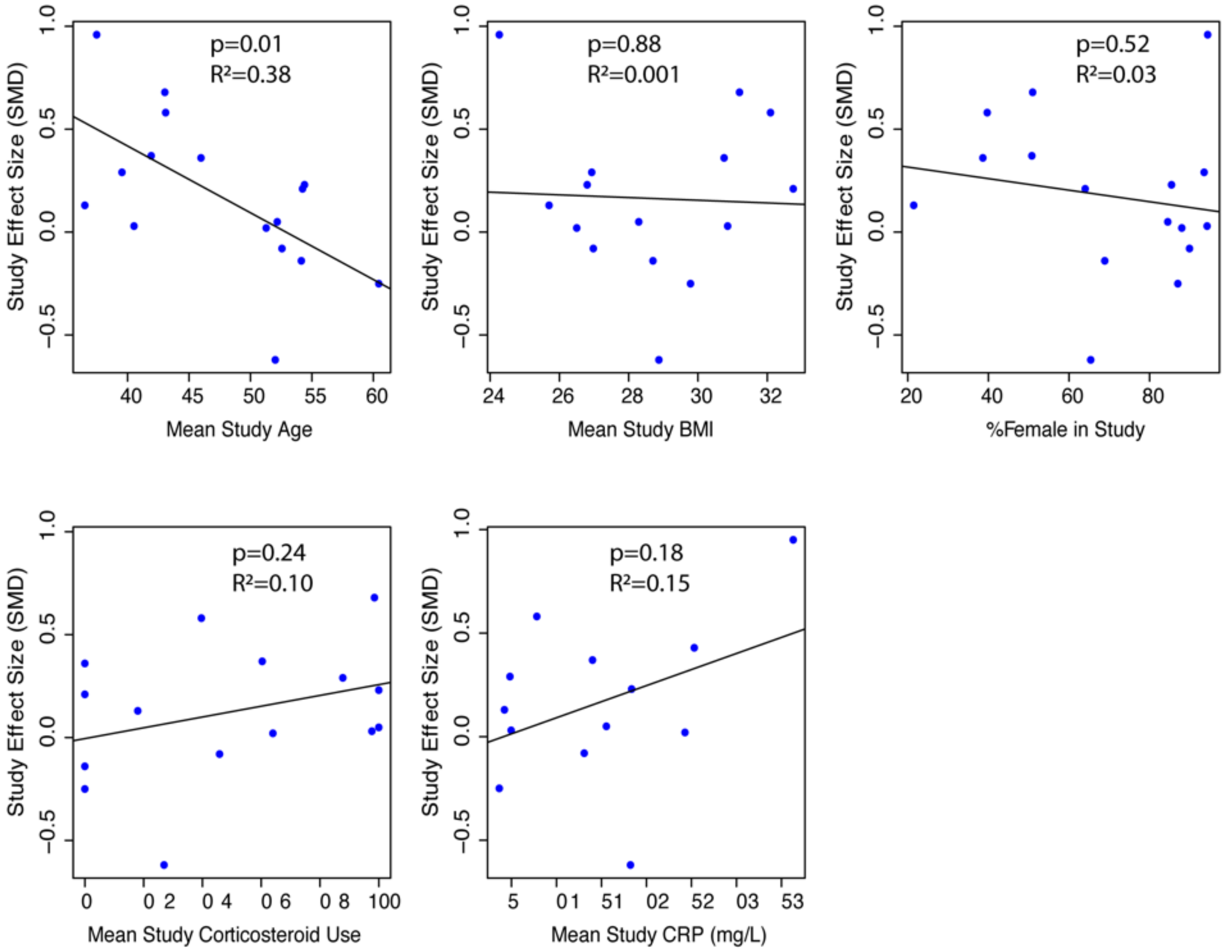

Supplementary Figure 8. Effect of mean study covariate values on overall study effect size on depressive symptoms. Each data point represents a single study. The y-axis indicates the Standardized Mean Difference (SMD) among patients with high depressive symptoms adjusted for primary disease symptom severity (as in Figure 2B), the x-axis corresponds to the mean value of the variable indicated within each study at baseline.

StudyID	ClinicalTrials.gov ID or Trial Publication	Study Drug	Treated Disease	Concomitant medications in placebo and treated arms
Janssen Trials				
**C0168T37**	http://www.nejm.org/doi/full/10.1056/NEJMoa050516#t=article	Infliximab	Ulcerative Colitis	Oral corticosteroids, 6 mercaptopurine, or azathioprine
**C0168T41**	http://onlinelibrary.wiley.com/doi/10.1002/art.21734/full	Infliximab	Rheumatoid Arthritis	Methotrexate
**C0168T44**		Infliximab	Psoriasis	None
**C0524T03**	NCT00207740	Golimumab	Asthma	Corticosteroids
**C0524T09**	NCT00265083	Golimumab	Ankylosing Spondylitis	None
**C0743T08**	NCT00267969	Ustekinumab	Psoriasis	None
**C0743T09**	NCT00307437	Ustekinumab	Psoriasis	None
**C1377T04**	NCT00718718	Sirukumab	Rheumatoid Arthritis	Methotrexate
**MCD2001**	NCT01024036	Siltuximab	Multicentric Castleman's Disease	Best Supportive Care
GlaxoSmithKline Trials				
**OFA110634**	NCT00603525	Ofatumumab	Rheumatoid Arthritis	Methotrexate
**OFA110635**	NCT00611455	Ofatumumab	Rheumatoid Arthritis	Methotrexate
**CXA30007**	NCT00120900	GW406381	Osteoarthritis-Knee	None
**CXA30009**	NCT00113308	GW406381	Rheumatoid Arthritis	None
**BEL110751**	NCT00410384	Belimumab	Lupus (SLE)	Standard SLE therapy*
**BEL110752**	NCT00424476	Belimumab	Lupus (SLE)	Standard SLE therapy*
**LBS02**	NCT00071487	Belimumab	Lupus (SLE)	Standard SLE therapy*
**KIP112967**	http://onlinelibrary.wiley.com/doi/10.1002/j.1532-2149.2012.00256.x/full	Losmapimod	Neuropathic Pain	None
**KIP113049**		Losmapimod	Neuropathic Pain	None
*Standard SLE therapies includes corticosteroids, anti-malarial agents, non-steroidal anti-inflammatory drugs, cytotoxic agents, immunosuppressive or immunomodulatory agents				

Supplementary Table 1: Additional clinical trial details. Reference IDs for all included clinical trials and details of the concomitant medications allowed per protocol as standard of care in placebo and treated arms. Informed consent was obtained from all subjects.

Study/Strata	Arm	Age	BMI	Sex (%Female)	Corticosteroid Use (%Yes)	Significant Covariates (HDS Adjusted)
**C0168T37**						None
High DS	Infliximab	39.4 (13.4)		50	57.1%	
	Placebo	47 (15)	-	52.4	61.9%	
Low DS	Infliximab	42.9 (14.7)		36.1	56.5%	
	Placebo	41.2 (13.2)	-	34.8	69.6%	
**C0168T41**						None
High DS	Infliximab	51.8 (12.4)	26.7 (5.2)	87.4	63.0%	
	Placebo	50.2 (11.8)	26 (6.1)	89.2	63.1%	
Low DS	Infliximab	52.5 (12.4)	27.1 (5.2)	76	56.5%	
	Placebo	53.2 (12.5)	27.5 (6.1)	83	57.7%	
**C0168T44**						None
High DS	Infliximab	42.5 (13.1)	31.3 (7.7)	55.8	2.6%	
	Placebo	44.6 (13.3)	30.9 (7.1)	36	0%	
Low DS	Infliximab	44.1 (12.5)	31.2 (7.1)	31.8	1.1%	
	Placebo	44.1 (12.1)	31 (6.6)	28.6	1.2%	
**C0524T03**						Age p=0.020
High DS	Golimumab	49.8 (13.8)	29.4 (6.7)	66.7	5.6%	
	Placebo	56.9(9.5)	27.7 (5.6)	62.5	12.5%	
Low DS	Golimumab	50.1 (12.3)	30.1 (7.4)	54.3	4%	
	Placebo	48.5 (12.3)	30.9 (8.6)	51.6	0%	
**C0524T09**						None
High DS	Golimumab	36.9 (10.5)	26.2 (5.7)	21.4	13.5%	
	Placebo	34.2 (9.6)	23.1 (4.4)	22.2	33.3%	
Low DS	Golimumab	39.3 (12.2)	26.4 (5.5)	27.1	19.2%	
	Placebo	41.1 (12.9)	27.1 (6.2)	29.8	16.4%	
**C0743T08**						Age p=0.041
High DS	Ustekinumab	41.5 (13.1)	32.4 (7.1)	38.7	4.5%	
	Placebo	46 (7.4)	31.5 (6.7)	42.1	0	
Low DS	Ustekinumab	45.8 (11.8)	31.7 (7.5)	30.8	1.5%	
	Placebo	44.8 (11.6)	31.4 (7.2)	26.6	1.7%	
**C0743T09**						None
High DS	Ustekinumab	45.4 (11.4)	30.5 (6.9)	41.2	1.4%	
	Placebo	47.3 (11.5)	31.2 (7.1)	33	0	
Low DS	Ustekinumab	46.1 (12.2)	30.8 (6.7)	28.8	0.7%	
	Placebo	46.9 (12.6)	30.42 (6.5)	29.5	1.7%	
**C1377T04**						None

High DS	Sirukumab	49.4 (11.1)	27.2 (4.7)	85.3	58.8	
	Placebo	49.3 (9)	28.6 (6.2)	91.7	66.7	
Low DS	Sirukumab	52.9 (10.9)	26.6 (5.7)	84.5	56.7	
	Placebo	51.6 (12.2)	26.5 (5.9)	63.6	45.5	
**MCD2001**						None
High DS	Siltuximab	44.8 (13.6)	25.5 (4.8)	45.5	18.2	
	Placebo	49.3 (14.0)	23.3 (3.4)	25	25	
Low DS	Siltuximab	43.8 (13.6)	24.4 (3.9)	42.5	32.5	
	Placebo	47.4 (13.6)	26.5 (5.5)	13.6	31.8	
**OFA110634**						None
High DS	Ofatumumab	52.3 (10.4)	26.7 (6.3)	89	100	
	Placebo	56.4 (10)	26.9 (4.6)	82	100	
Low DS	Ofatumumab	53.9 (15.1)	25.2 (4.6)	84	100	
	Placebo	51.8 (12.4)	26.7 (4.8)	80	100	
**OFA110635**						None
High DS	Ofatumumab	52.7 (9.5)	29 (6.3)	86	100	
	Placebo	51.7 (11)	27.6 (4.3)	83	100	
Low DS	Ofatumumab	51.4 (11.9)	27.8 (5)	79	100	
	Placebo	54 (11.4)	27.4 (5.1)	83	100	
**CXA30007**						None
High DS	GW406381	60.3 (9)	30.4 (6.2)	87	0	
	Placebo	61 (10.9)	27.4 (3.9)	87	0	
Low DS	GW406381	60.3 (9.4)	30.7 (6.5)	67	<1	
	Placebo	60.1 (9.9)	29.7 (5.2)	71	<1	
**CXA30009**						None
High DS	GW406381	52.9 (11.6)	27 (5.7)	91	47	
	Placebo	51.4 (11.3)	26.9 (5.9)	86	42	
Low DS	GW406381	52.7 (11.8)	26.8 (5.4)	82	39	
	Placebo	52.3 (11.6)	26.4 (4.8)	87	36	
**BEL110751**						None
High DS	Belimumab	38.6 (9.3)	27 (5.5)	96	86	
	Placebo	41.3 (11)	26.8 (6)	89	91	
Low DS	Belimumab	40.5 (11.6)	27.4 (7.9)	94	83	
	Placebo	39.8 (12.1)	26.5 (6)	93	85	
**BEL110752**						None
High DS	Belimumab	36 (10.9)	24.2 (4.8)	97	98	
	Placebo	40.6 (11.9)	24.4 (4.7)	89	96	
Low DS	Belimumab	35 (10.7)	24 (4.7)	95	97	

	Placebo	35.3 (11.6)	24.1 (4.6)	95	96	
**LBS02**						None
High DS	Belimumab	40.6 (8.8)	30.4 (8.3)	93	84	
	Placebo	40.2 (10.8)	32.9 (10.9)	100	80	
Low DS	Belimumab	42.2 (11.6)	29.5 (7.8)	94	78	
	Placebo	42.4 (10.9)	30.7 (7.2)	89	83	
**KIP112967**						None
High DS	Losmapimod	50.9 (15.9)	26.4 (7.4)	64	0	
	Placebo	57.6 (10.1)	29.1 (5.4)	64	0	
Low DS	Losmapimod	52.6 (13.9)	28.5 (6.2)	64	0	
	Placebo	49.9 (13.3)	26.9 (4.7)	61	0	
**KIP113049**						None
High DS	Losmapimod	57.3 (10.2)	28 (3.3)	89	0	
	Placebo	50.1 (5.3)	29.6 (6.1)	43	0	
Low DS	Losmapimod	58.7 (11.2)	29 (4.5)	58	2	
	Placebo	59.3 (11)	28.9 (5.1)	48	0	

Supplementary Table 2: Patient demographics and concomitant corticosteroid use for each trial. We report demographic information on age, body mass index (BMI), sex, and corticosteroid use for each trial, stratified by treatment and high depressive symptom cutoff. We report the significance in the statistical model fitting age, bmi, sex and corticosteroid use for each covariate among patients with high depressive symptoms as described in the Supplementary Methods.

	Clinical Trial Accession Number	High Depressive Symptoms		Low Depressive Symptoms Mean(SD)		P-Value (Wilcoxon)	Treatment Spearman ρ (p-value)	Placebo Spearman ρ (p-value)
		**Mean±SD**	**N**	**Mean±SD**	**N**			
**CRP (mg/L)**	C016T37	16.0 ± 25.3		13.5 ± 18.5		0.9	-0.03 (0.88)	0.11 (0.65)
	C0168T41	24.9 ± 29.0		24.1 ± 24.0		0.6	-0.04 (0.66)	**0.33 (0.008)**
	C0524T03	21.5 ± 23.6		17.5 ± 19.4		0.3	0.10 (0.50)	-0.29 (0.49)
	C0524T09	3.81± 3.97		4.27± 4.61		0.9	0.23 (0.44)	NA
	C0743T08	**15.4± 34.2**		**7.1± 9.1**		**0.03**	0.01 (0.95)	-0.01 (0.98)
	C0743T08	**10.1± 18.5**		**7.1± 11.8**		**0.003**	-0.07 (0.33)	-0.03 (0.80)
	C1377T04	26.2 ± 24.5		25.0 ± 21.1		0.9	-0.23 (0.21)	0.01 (0.98)
	MCD2001	**66.1 ± 62.6**		**29.1 ± 41.2**		**0.03**	-0.38 (0.25)	0.11 (0.89)
**TNF-α (pg/ml)**	C0524T03	5.21± 3.38		4.42± 2.13		0.35	0.18 (0.47)	NA
**IL-6 (pg/ml)**	C0524T03	4.08± 4.38		4.06± 4.70		0.97	0.15 (0.54)	NA
	C1377T04	25.6 ± 37.3		25.2 ± 62.3		0.52	-0.13 (0.49)	-0.12 (0.75)
	MCD2001	9.5 ± 7.6		7.6 ± 8.3		0.18	-0.33 (0.36)	0.63 (0.37)

*hs-CRP. C524T03, placebo arm too small (N=2) for correlation analysis of TNF-α and IL-6.

Supplementary Table 3: Baseline biomarkers. Left: Baseline biomarkers are shown for high depressive symptom and low-depressive symptom cohorts. Right: For patients with high depressive symptoms at baseline, the partial Spearman correlation between baseline biomarker value and change in depressive symptoms, corrected for change in primary disease symptom severity. *P*-values are two-sided.
